# Topography measurements and applications in ballistics and tool mark identifications^*^

**DOI:** 10.1088/2051-672X/4/1/013002

**Published:** 2015-12-17

**Authors:** T V Vorburger, J Song, N Petraco

**Affiliations:** 1 National Institute of Standards and Technology (NIST), Gaithersburg, MD 20899, USA; 2 City University of New York, John Jay College and The Graduate Center, New York, NY 10019, USA

**Keywords:** ballistics, breech face, bullet, cartridge case, firearm, surface, standards

## Abstract

The application of surface topography measurement methods to the field of firearm and toolmark analysis is fairly new. The field has been boosted by the development of a number of competing optical methods, which has improved the speed and accuracy of surface topography acquisitions. We describe here some of these measurement methods as well as several analytical methods for assessing similarities and differences among pairs of surfaces. We also provide a few examples of research results to identify cartridge cases originating from the same firearm or tool marks produced by the same tool. Physical standards and issues of traceability are also discussed.

## 1. Introduction

The field of firearms identification is more than 100 years old and the field of surface topography measurement is at least 80 years old, but the combination of the two, surface topography measurements and analysis in the study of fired cartridge cases and bullets, has only been around for about 15 years. The combined field is so new that firearm identifications at crime labs using the cartridge case or bullet impressions are still performed manually by experts in optical (side-by-side) comparison microscopes. Nevertheless, the field has a promising future with a vision that one day investigations and firearm identifications might be accomplished or affirmed through automated searches and matches using topography data from the surfaces of the ballistics evidence.

This review describes some of the methods practiced and results accomplished thus far in the field. Gerules *et al* published a broad review of methods for firearms analysis in 2010 [1]. The current review focuses on topography methods with some illustrative examples and on recent work. The remainder of section 1 provides a few highlights of the history of firearms identification. Section 2 describes surface topography measurement and section 3 describes analysis procedures and parameters, especially those to quantify similarity between surface topography images. Section 3.7 discusses the all-important issue of error rate estimation. Section 4 describes standards, notably physical standards, documentary standards, and uncertainty and traceability issues. Examples of work in the field are interspersed thoughout but additional results are given in section 5. Section 6 discusses applications of these methods in the wider field of surface metrology. Section 7 highlights a few ongoing issues and opportunities.

### 1.1. Firearm and tool mark identifications

**Figure 1:**
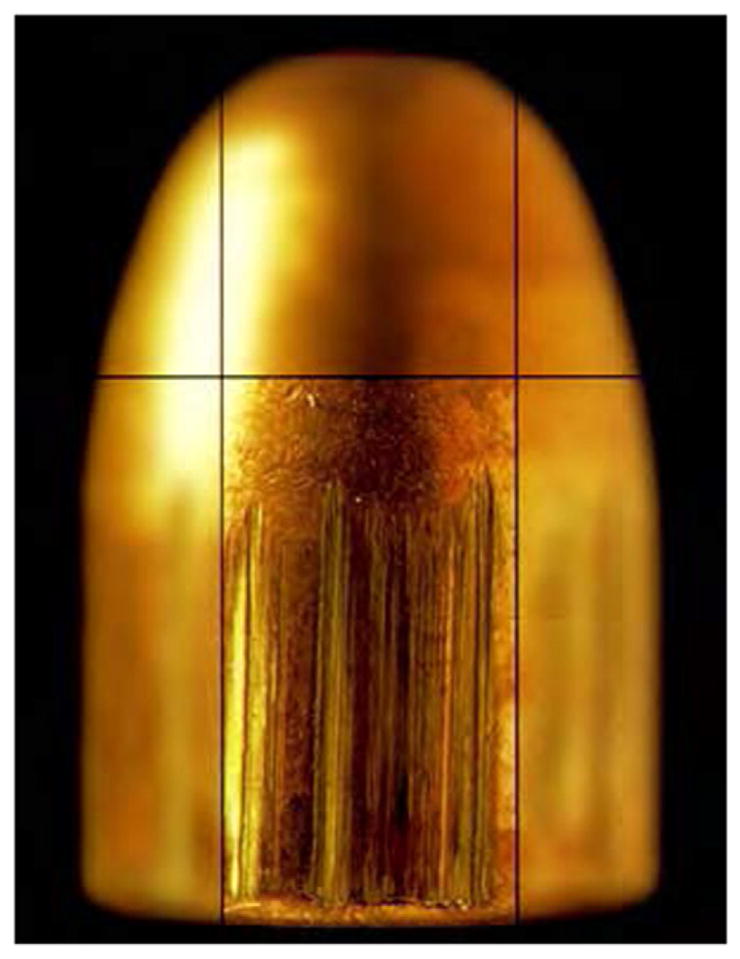
Striations on one of several land engraved areas (LEAs) on a fired bullet. The widely used 9 mm caliber firearms engrave bullets with six LEAs (Courtesy of Ultra Electronics Forensic Technology, Inc. [3]).

**Figure 2:**
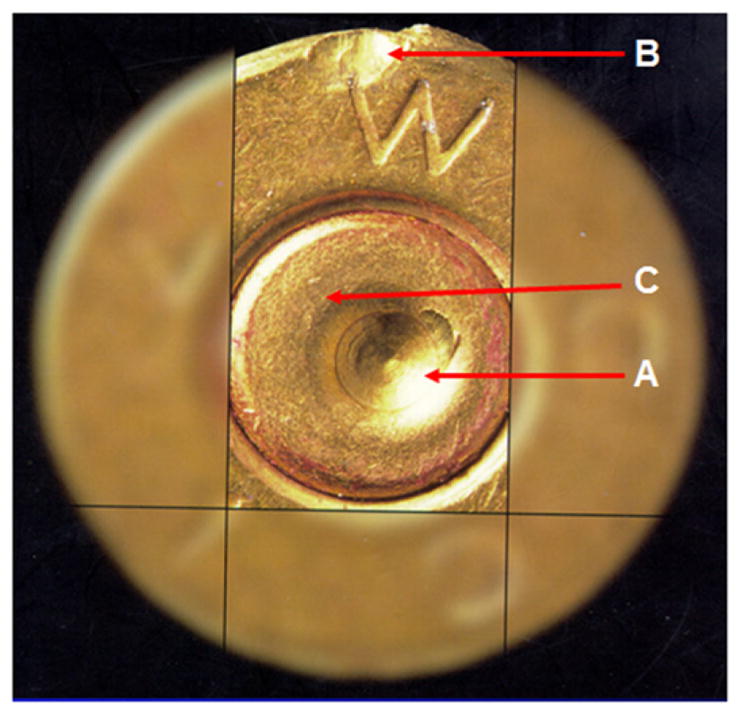
Firearm signatures on a fired cartridge case include the firing pin impression (A), the ejector mark (B), and the breech face impression (C) (Courtesy of Ultra Electronics Forensic Technology, Inc. [3]).

Tool marks are permanent changes in the topography of a surface created by forced contact with a harder surface (the tool). When bullets are fired and cartridge cases ejected from a firearm, the parts of the firearm that make forcible contact with them create characteristic tool marks on their surfaces called ‘ballistic signatures’ [2]. Striation signatures (2D profile tool marks) on a bullet are caused by its passage through the gun barrel (see figure 1 [3]). Impression signatures (3D topography tool marks) on a cartridge case are caused by impact with the firing pin, breech face and ejector (see figure 2 [3]). Both the 2D striation and 3D impression signatures are unique to the firearm. By microscopically comparing these ballistic signatures, firearm examiners can determine whether a pair of bullets or cartridge cases was fired or ejected from the same firearm. Ballistics examiners can then connect a recovered firearm or other firearm evidence to criminal acts.

**Figure 3:**
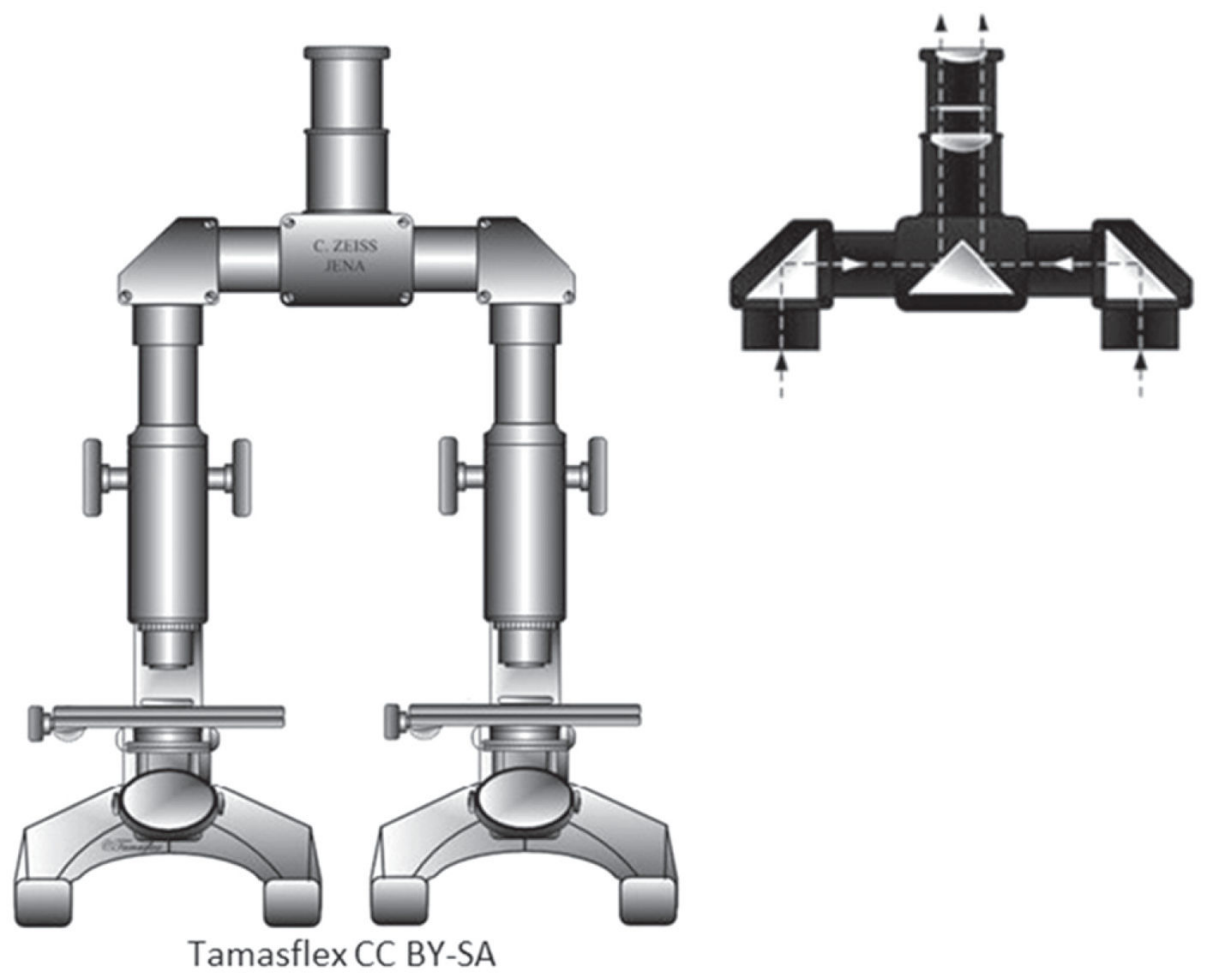
The two-ways comparison microscope invented by Alexander von Inostranzeff in 1885 [5] has been widely used for ballistics image comparisons since the 1930s (illustration from Wikipedia [8]; see also Zheng *et al* [9]).

**Figure 4:**
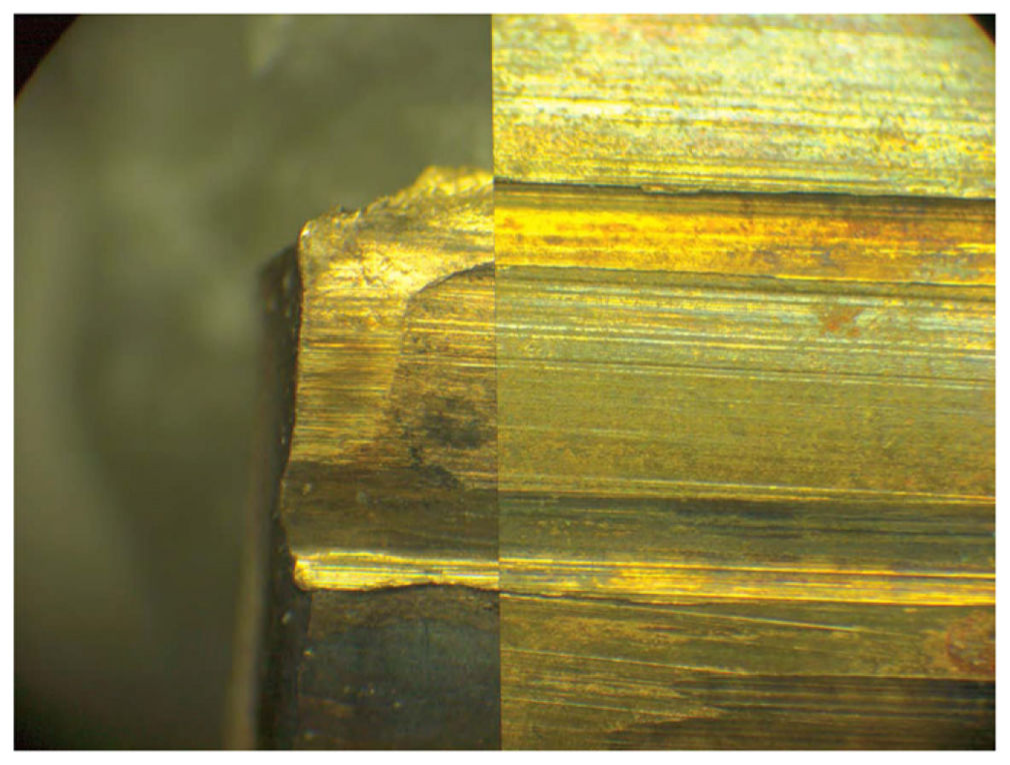
Split image in a comparison microscope of a bullet fragment (left) and a bullet test fired from a suspect firearm right (with permission of the National District Attorney’s Association [7]).

**Figure 5:**
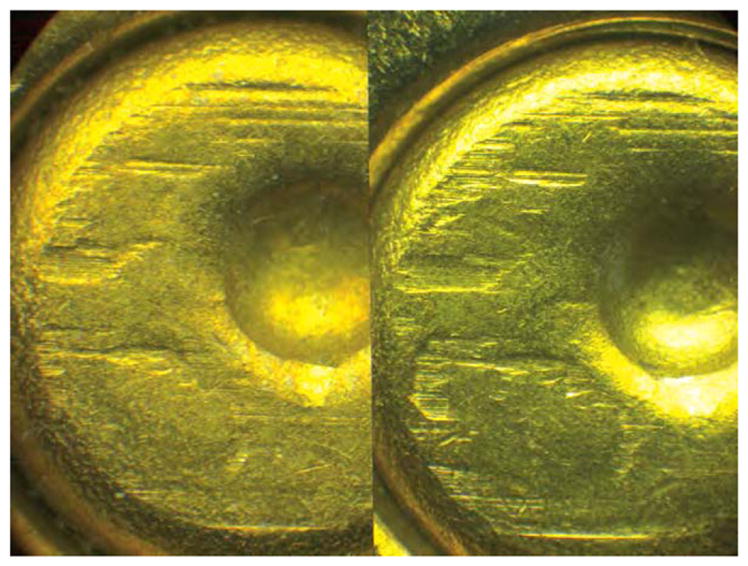
Microscopic comparison of breech face detail on two cartridge cases (with permission of the National District Attorney’s Association [7]).

Side-by-side tool mark image comparisons for ballistic identification have more than a hundred year history [4]. The earliest known firearm evidence identification for investigating a crime case dates from 1835 in London, England. Henry Godda, a Bow Street Runner (an early police force in London) was able to identify the mold mark on a fired projectile (ball) [4] produced by the mold used to form it from molten lead. However, it was not until the early twentieth century that firearms identification came into its own as a science, when the ‘two-way comparison microscope’ invented by Alexander von Inostranzeff in 1885 [5] (figure 3) was adapted for firearm identification and research [6]. Since the 1930s, the use of comparison microscopes for firearm evidence identifications [4] improved ballistics image comparisons by displaying the reference image and the evidence image side-by-side, and shifting them relative to one another to optimize the comparison. Furthermore, the microscope can capture both the reference and the evidence images under the same lighting conditions (or nearly so), an important issue for comparison of optical images. Figures 4 and 5 show typical side-by-images of the striations on a pair of bullets and cartridge cases [7], respectively, apparently fired by the same gun.

Since the early 1990s, commercial automated ballistics identification systems, such as the Drugfire [10] and the Integrated Ballistics Identification System (IBIS) [11], have been developed, producing a revolution in the speed at which microscope inspections can proceed. Such systems typically include a digitized optical microscope to acquire images of bullets and cartridge case surfaces, a signature analysis station, correlation software, and access to a large database where accumulated images reside. With such a system a large number of comparisons can be performed automatically. When a suspect image is input into the database, it is correlated with the images in the database, and a list of possible leading matches (say, top 10) is output for further analysis by firearms examiners, who can directly verify a match by comparison of the original materials in a comparison microscope.

Most of these systems are based on comparisons of the optical intensity images acquired by the microscope. The quality of optical images is largely affected by lighting conditions such as the type of light source, lighting direction, intensity, color and reflectivity of the material, and the image contrast. Since each of the images is acquired alone and not in a comparison microscope, the systems are more susceptible to slight variations in the alignment and lighting conditions. The significant effect of lighting conditions on the optical image has been discussed by Song *et al* [12], and Chu *et al* [13].

Accurate identification also depends on the capability of the correlation software to identify the related correlation regions and to eliminate the unrelated regions from correlation. Current commercial systems use proprietary correlation parameters and algorithms to quantify image similarity. These proprietary correlation methods lack objective open tests of their parameters and algorithms and hence lack metrological traceability. This may pose difficulty for laboratory assessments and inter-comparisons among different systems.

Ballistics signatures are 2D or 3D tool marks and therefore, must be geometrical surface topographies by nature. It was stated in the ‘*Theory of Identification*’ issued by the Association of Firearm and Tool mark Examiners (AFTE) that ‘…*the comparison of tool marks*…’ are to be made on the ‘…*unique surface contours*…’ and ‘*surface contour patterns comprised of individual peaks, ridges and furrows. Specially, the relative height or depth, width, curvature*…’ [2, 4]. Because ballistics signatures are geometrical micro-topographies by nature, direct measurement and correlation of the 2D surface profiles and 3D surface topographies have been proposed for ballistic identification [14–16]. Such methods can avoid the confusing effects of variable lighting conditions and shadowing, and should likely improve correlation accuracy of automated systems. Since the 1980s, with the help of modern computer technology, several different types of optical instruments have been developed, which are capable of precise measurement of surface topography. These methods will be discussed in section 2. They are making it possible to use quantitative topography measurements for firearm evidence identifications, in addition to traditional methods based on conventional image comparisons. Development of ballistics identifications is therefore facing a likely evolution from qualitative image comparisons to quantitative topography measurements [15].

## 2. Topography measurement

Generally speaking, geometrical ballistics signatures fall into two categories: bullet signatures consisting of striations that can be represented by 2D profiles, *z*(*x*); and impression signatures on different regions of the cartridge cases, including firing pin, breech face and ejector mark signatures that can be represented by 3D topography images *z*(*x*, *y*). Conventional optical intensity images, *I*(*x*, *y*) have also been referred to as 2D. However, in this review we will confine the term 2D to height profiles *z*(*x*).

**Figure 6:**
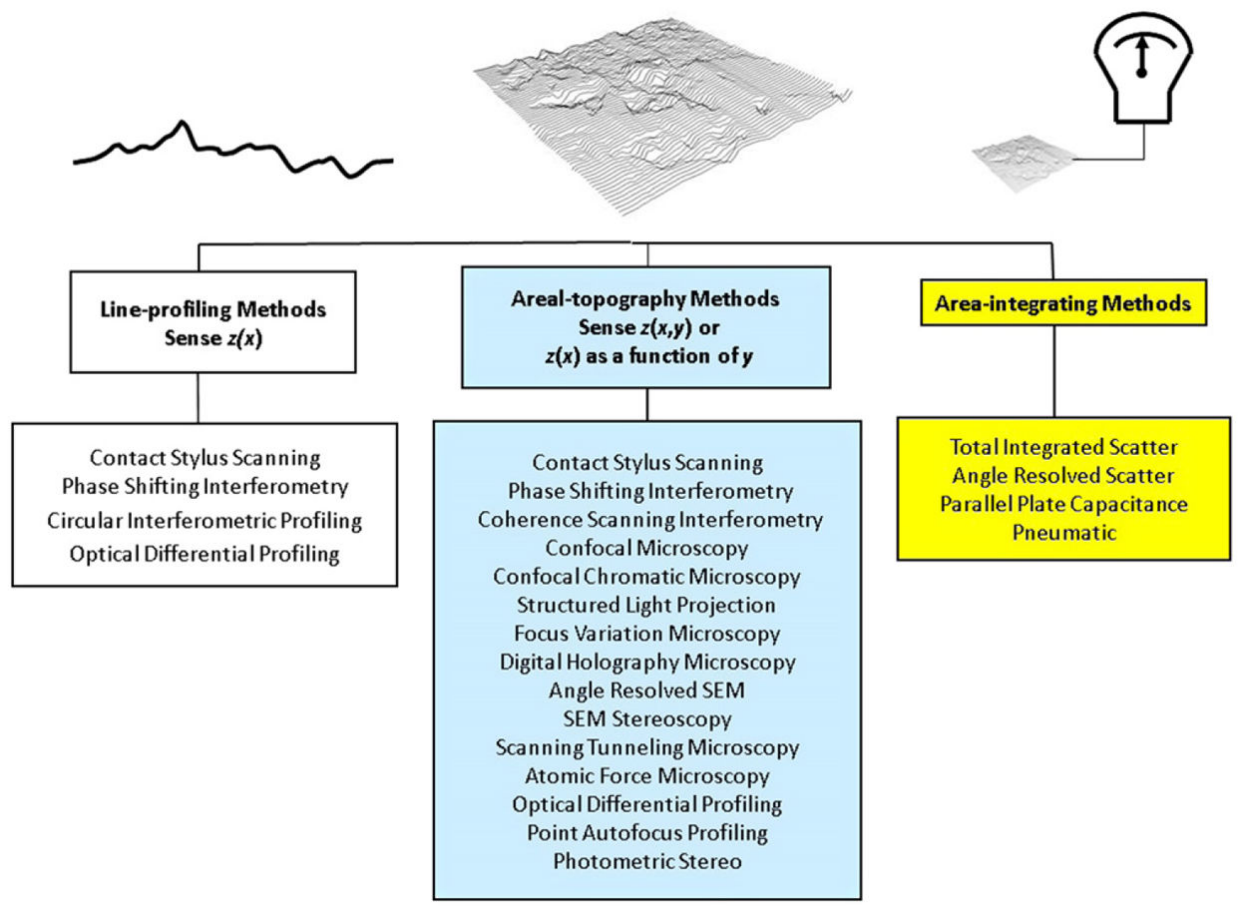
A classification of surface texture measurement methods with examples (see also earlier versions [17, 18].

A number of different methods have been developed to measure surface topography. They may first be classified into three categories—line-profiling, areal-topography, and area-integrating—as described in an ISO standard (see figure 6) [17, 18]. In this review we will emphasize line profiling and areal topography. In both methods, surface profiles or topography images, represented mathematically as *z*(*x*) or *z*(*x*, *y*) respectively, are developed by probing the surface heights with high lateral resolution. The third category, area-integrating, where a single measure of surface texture over an area is estimated by probing the entire area at once, to our knowledge has not been used for firearms research or identification.

The lateral range capability of profiling and topography instruments varies widely depending on the application—from kilometers, the case for highway profilers, to submicrometers, the case for atomic force microscopes. The vertical range, vertical resolution, and lateral resolution of these instruments roughly scale with the lateral range. For people using road profilers, surface features of interest may be some millimeters high and several hundred millimeters wide, whereas for people using atomic force microscopes, features of interest may be subnanometers high and nanometers wide. The surface topography features of cartridge cases and bullets, which are of interest to firearms experts, are generally in the micrometers to millimeters lateral range with heights in the sub-micrometer to hundred micrometer range. Even in this relatively narrow range there are at least five different methods, most of them optical, which are useful and available as commercial instruments. The following subsections describe them. Most of these methods are the subject of international documentary standards that outline the key properties and describe influence factors that are potential sources of uncertainty and error. Calibration procedures are currently the subject of further standardization efforts.

### 2.1. Stylus instrument

**Figure 7:**
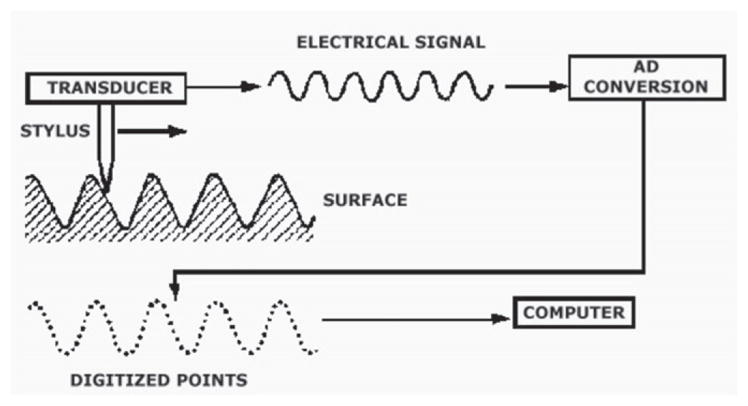
Schematic of stylus instrument for measuring surface topography [21].

Stylus instruments [19, 20], produce surface profiles and topography images by scanning the surface with a fine stylus (figure 7) [21]. As the stylus scans the surface, its vertical motion over the peaks and valleys is converted by a transducer into an electrical signal that is digitized, stored, and analyzed. Stylus instruments can have a very good range-to-resolution ratio in both the vertical and lateral directions and can have high accuracy once calibrated. The fact that they require mechanical contact limits their utility for inspection of the surfaces of evidence materials or test fired ammunition because of the potential for scratching or otherwise damaging the surfaces under inspection. Stylus instruments have been used effectively in some firearms and tool mark research [22] and in measurements of physical standards for bullets [23].

The large majority of instruments used for measuring the topography of ballistics and toolmarks are optical. We will discuss these over the next several subsections.

### 2.2. Confocal microscopy

**Figure 8:**
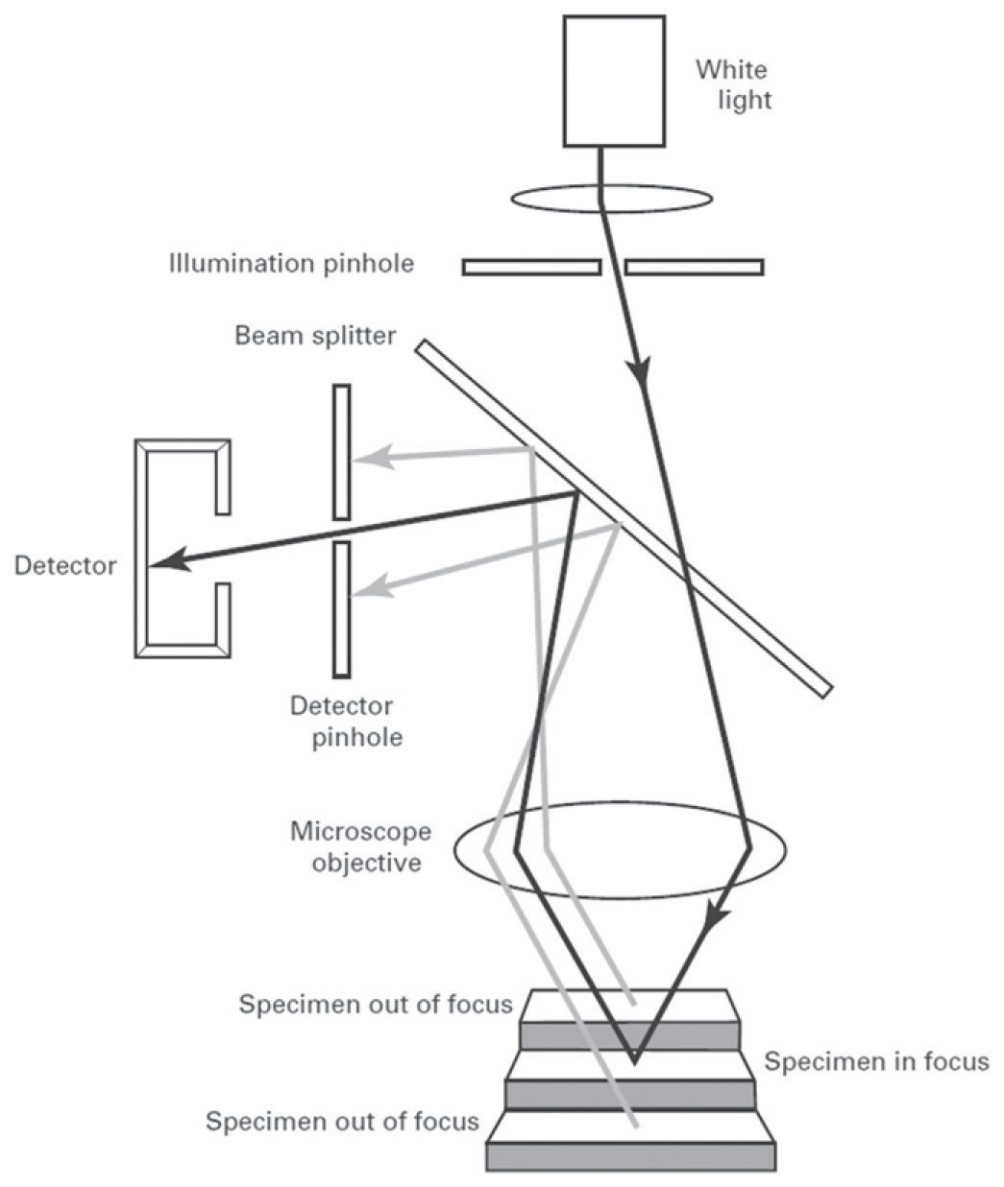
Schematic diagram of a confocal microscope for measuring surface topography (reprinted with permission) [26].

**Figure 9:**
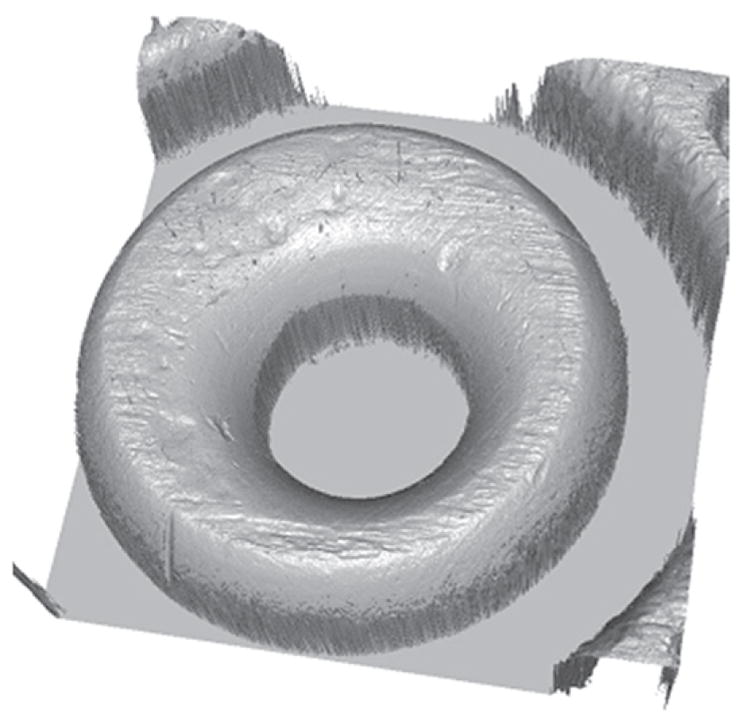
Topography image of the breech face impression of a fired 9 mm cartridge case obtained with disk scanning confocal microscopy [16]. The field of view is roughly 4 mm on a side.

**Figure 10:**
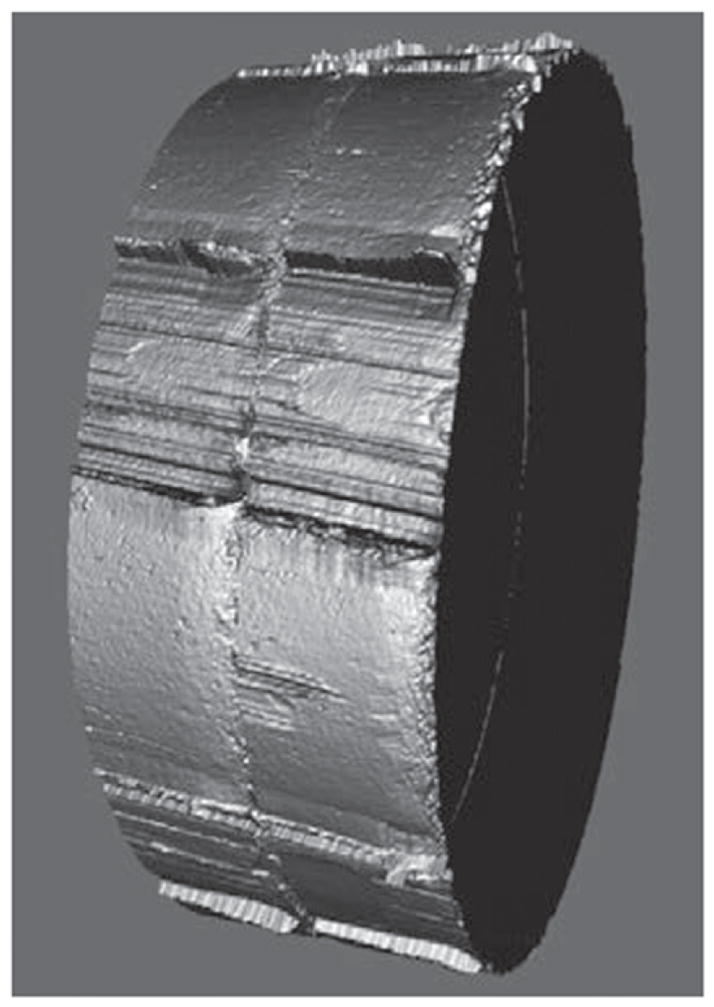
Topography image of 360° bands of two fired 9 mm caliber bullets obtained with disk scanning confocal microscopy. For each image, the system captured and stitched 1.6 mm × 1.6 mm areas while rotating and translating the bullet (courtesy of Ultra Electronics Forensic Technology, Inc. [3]).

Confocal microscopy [24] is widely used, not only for fluorescence microscopy and 3D sectioning of transparent materials, but for the measurement of surface topography when used in reflection mode. A working draft standard [25], which describes confocal microscopy and its influence quantities, has recently undergone an ISO ballot as a New Work Item. A schematic diagram of a typical confocal microscope is shown in figure 8 [26]. Most examples of this method rely on the use of pinholes for height discrimination. Incident light is focused through a pinhole, refocused onto the surface and reflected from it, then refocused through a conjugate pinhole placed before the detector. A strong signal through the pinhole will be detected only when the surface point is at the focusing height. This discrimination enables the tool to detect variations in surface height and topography when the surface is vertically scanned (figure 8) along the optical axis of the microscope. Variations in the method include laser scanning confocal microscopy (LSCM), disc scanning confocal microscopy (DSCM), and programmable array microscopy (PAM) [25, 27]. The latter method uses switchable elements in a programmable array to define a tiny light source instead of pinholes themselves. Different confocal microscopes have been used in a number of firearms and tool mark research studies [16, 28–31]. The vertical noise resolution and lateral resolution improve with the numerical aperture (NA) of the microscope. With a 50X objective, having a NA of 0.5, the vertical resolution can reach a few nanometers and the lateral resolution is on the order of a micrometer or less. A topography image of a fired cartridge case obtained with confocal microscopy is shown in figure 9 [16]. A topography image of two compared bullet sections obtained by confocal microscopy is shown in figure 10 [3].

### 2.3. Coherence scanning interferometry (CSI)

**Figure 11:**
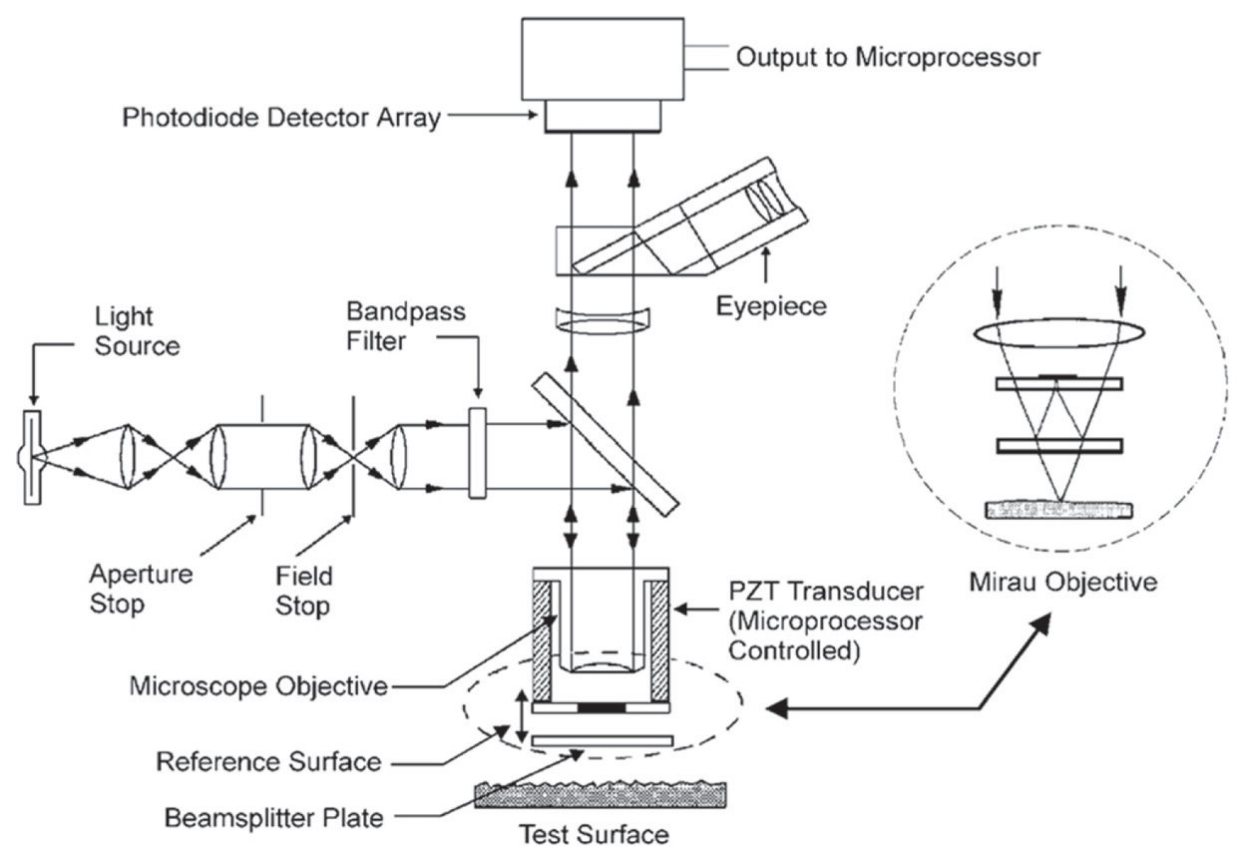
Schematic diagram of a coherence scanning interferometric microscope in the Mirau configuration (reprinted with permission) [34].

**Figure 12:**
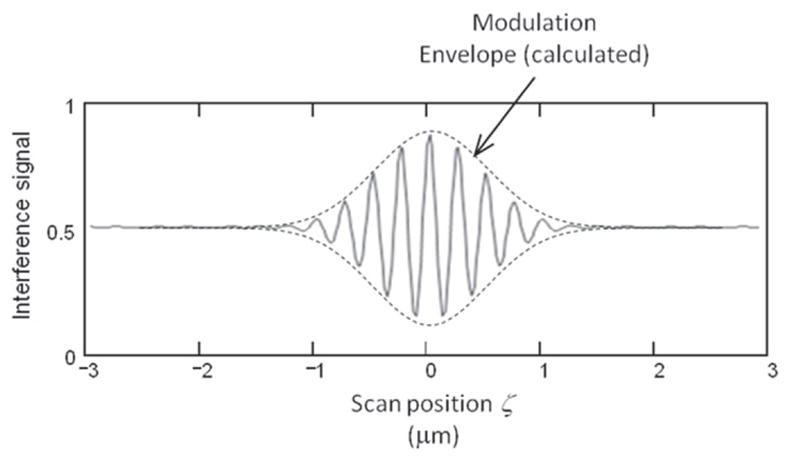
Schematic diagram [33] of the modulation signal for CSI for a single pixel (With permission of Springer Science + Business Media).

**Figure 13:**
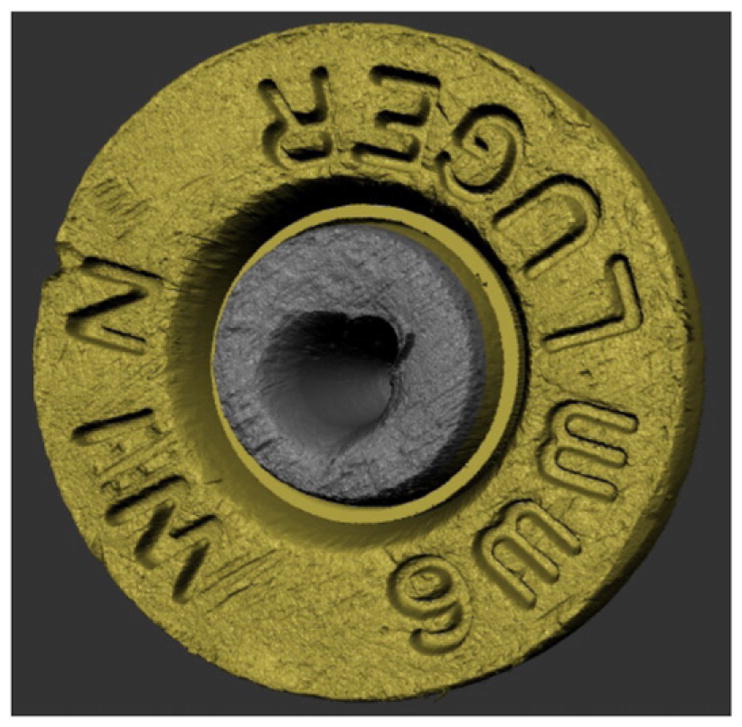
Topography image of a fired cartridge case obtained with a method closely related to CSI (reprinted with permission) [35].

CSI relies on interference between a beam of light reflected from the surface under study and a beam of light reflected from a reference surface. This method is the subject of a published standard [32] and other reviews [33]. A schematic diagram is shown in figure 11 [34]. When the optical paths reflected from the reference surface and the test surface are equal, an interference pattern of bright and dark fringes is formed on the camera detector, but as either optical path is changed by distances larger than the coherence length of the light, the fringe contrast disappears. For any single pixel, one expects a fringe pattern like that shown in figure 12 [33]. One can move the surface or the microscope vertically to observe a maximum in the signal modulation in order to locate the height of a surface point relative to its neighboring points. Alternatively, transform algorithms [33] have been developed to locate the surface height from data like that shown schematically in figure 12. The vertical noise resolution is routinely a few nanometers but under some conditions can be as small as about 0.1 nm. The lateral resolution scales with the NA of the microscope in a manner similar to the confocal method. A topography image of a fired cartridge case obtained by an interferometric method closely related to CSI is shown in figure 13 [35].

Phase shifting interferometry [36] is another form of interference microscopy, which has even higher vertical resolution than CSI but limited vertical range and has, so far, not proven useful for measuring the rough surfaces of fired cartridge cases and bullets.

### 2.4. Focus variation

**Figure 14:**
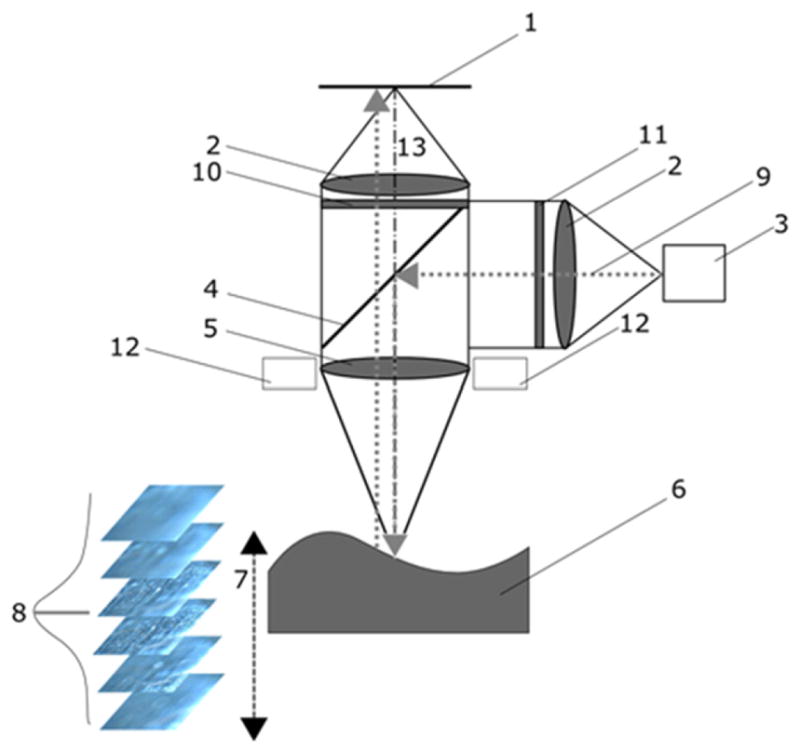
Schematic of a focus variation microscope [38]. (1) Camera sensor, (2) lenses, (3) light source, (4) semi-transparent mirror, (5) objective lens with limited depth of field, (6) sample, (7) vertical movement with drive unit, (8) contrast curve calculated from the local window, (9) light rays from the white light source, (10) analyzer, (11) polarizer, (12) ring light. Items 10–12 are optional (©ISO. This material is reproduced from ISO/FDIS 25178-606:2014 with permission of the American National Standards Institute (ANSI) on behalf of ISO. All rights reserved.).

**Figure 15:**
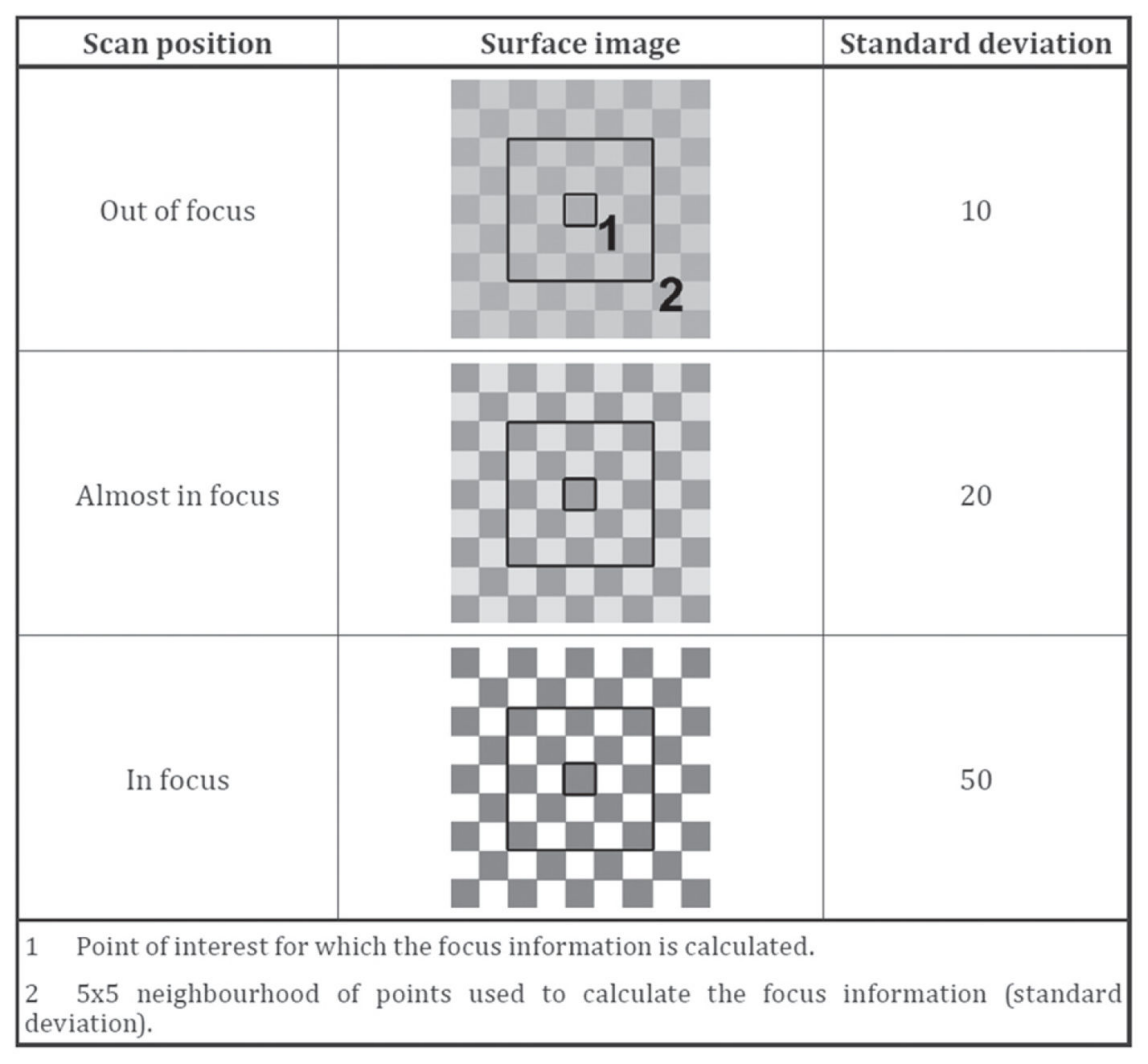
Calculation of focus information at a position of interest (1) using the contrast from a neighborhood of points (2). The contrast may be quantified by the standard deviation of the intensities of the neighboring points (©ISO. This material is reproduced from ISO/FDIS 25178-606:2014 with permission of the American National Standards Institute (ANSI) on behalf of ISO. All rights reserved. ISO/FDIS 25178-606:2014 is an ISO draft document that is subject to change without notice. It cannot be referred to as an approved ISO standard) [38].

Both confocal and CSI methods involve some manipulation of the light traveling through a microscope, either with pinholes or beam splitters. This leads to a cost in signal-to-noise. Focus variation [37] (figure 14) is conceptually simpler. The height sensing function derives from locating the surface at its sharpest, best focus position in the microscope. The peaks and valleys of the surface are focused at different positions as the surface scans vertically with respect to the microscope, a mode of operation similar to those of confocal and CSI. Focus variation is the subject of a Final Draft International Standard [38]. The method is capable of measuring steeply sloped surfaces, up to nearly 90° [37]. Because the method relies on contrast in images resulting from peaks and valleys of surface features, averaging of individual pixels is required to provide the height sensitivity, which involves a collective response from neighboring pixels as illustrated in figure 15 [38]. This implies that both the lateral resolution and vertical resolution of the focus variation method are more limited than those for confocal or coherence scanning. Therefore, a question arises requiring further research as to whether the straightforward method of focus variation has sufficient resolution for distinguishing the individualized surface characteristics of fired bullets and cartridge cases. Along this line, focus variation has been favorably reviewed by Bolton-King *et al* [39].

### 2.5. Photometric stereo

**Figure 16:**
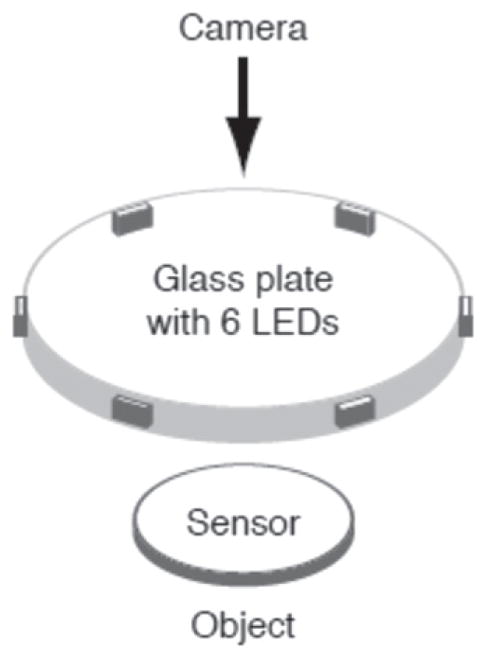
Schematic detail of a photometric stereo tool known as Gelsight [40] for measuring surface topography. Six LED light sources illuminate the rough surface of the object in turn at near grazing incidence angle. The sensor is a soft material with uniform optical properties that replicates the rough surface topography of the object when pressed down against it. The microscope between the glass plate and the camera is not shown (© 2011 Association for Computing Machinery, Inc. reprinted by permission) [40].

**Figure 17:**
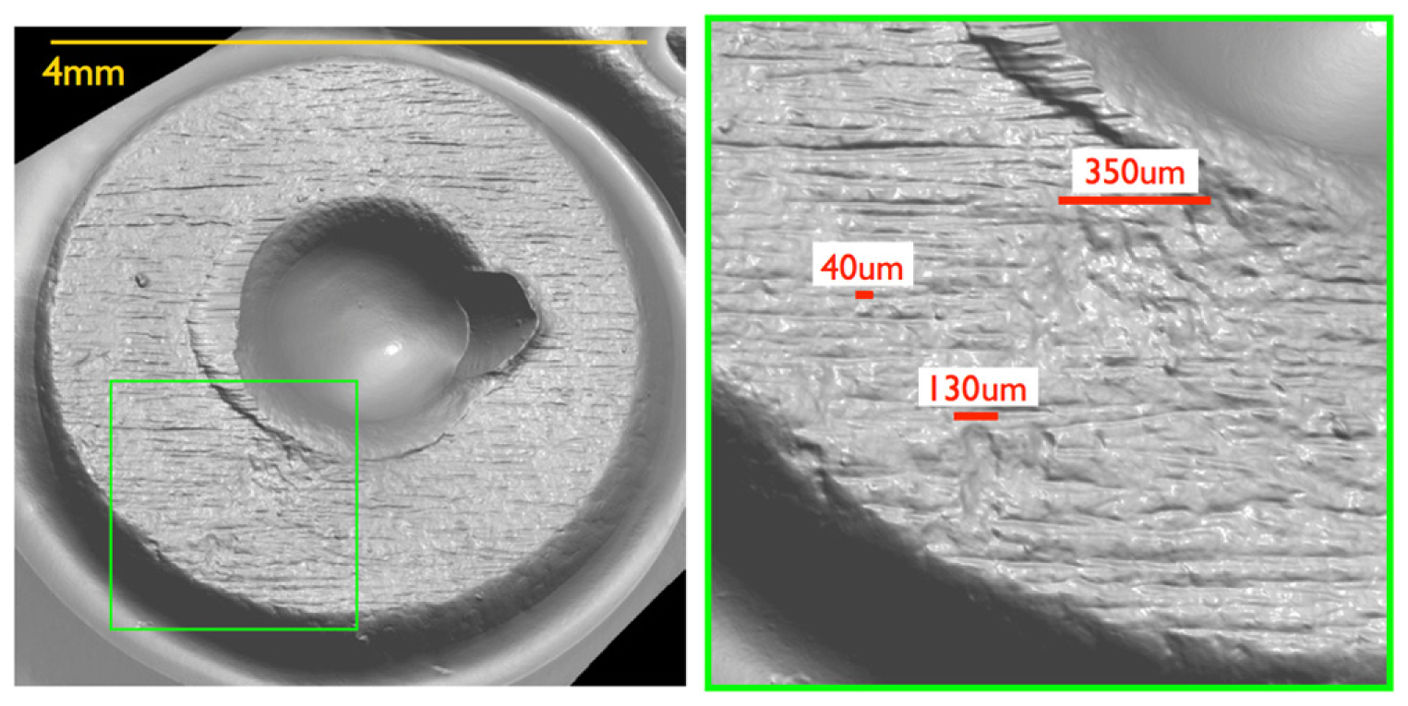
Topography image of the breech face impression of a unit of SRM 2461 Cartridge Case obtained with a photometric stereo tool (Originally published by the National Institute of Justice, US Department of Justice) [42].

Photometric stereo, also called shape from shading, involves the decoding of shadow patterns on surfaces cast by multiple light sources to produce a surface topography measurement. Depending on the number and directions of the light sources, this method can have different manifestations [40, 41]. One of these is shown in figure 16 [40]. Six light sources evenly spaced azimuthally illuminate the surface in turn at a grazing angle. The shadow patterns are analyzed and produce a surface topography image. The method illustrated here includes an additional technique, called Gelsight, to reduce the sensitivity to variations in surface optical properties and to emphasize the surface topography. Integral to the setup is a soft, transparent gel with a gray film that directly contacts the surface. The gray film has uniform optical properties and a small grain size, which helps to diffuse the reflected light to minimize confusing highlights. The microscope above the gel observes the shadows of the gel surface and the gel surface closely reproduces the underlying topography itself. A topography image of the breech face impression of a unit of National Institute of Standards and Technology (NIST) Standard Reference Material (SRM) 2461 obtained with photometric stereo is shown in figure 17 [42].

The photometric stereo method may be less expensive and more convenient to use than other methods, but its resolution is not expected to be as high as confocal microscopy or CSI. Researchers and firearms examiners, therefore, face an interesting research issue: do the methods, which are likely simpler or less expensive, photometric stereo and focus variation, have sufficient lateral and vertical resolution to perform as well as the high resolution methods, confocal and CSI, in the task of measuring individualizing topography features important to firearms investigations? What lateral resolution is needed: 1 *μ*m? A few micrometers? Or is 10 *μ*m good enough?

## 3. Analysis and parameters

### 3.1. The importance of similarity as a surface property in this field

The function of establishing whether or not two bullets or cartridge cases were fired by the same gun depends on obtaining some assessment of similarity between them. The key surface topography function to be quantified is not a complex phenomenon, like sliding friction between two surfaces or the propensity for wear of one of them. It is not even the relative diffuseness of specularity of the surface as a light scatterer. The surface function we want to quantify is simply the degree of similarity of a pair of surfaces. Can one derive a measure of similarity of two surfaces that will lead to identification or exclusion of them as being fired by the same firearm. To accomplish this task, the firearms examiner applies his/her expert judgment in a way that is difficult to quantify. An automated system, by contrast, must be programmed to produce a quantitative measurand for similarity, which the expert can use. Hence, much research in firearms identification is concentrated on finding algorithms and parameters that emphasize the individualized characteristics of surfaces and their similarity to those of other surfaces. Two ways [43] to do this are to identify individual features on one member of a pair and look for similar features on the other or to match a large section of one surface to that of the other.

**Figure 18:**
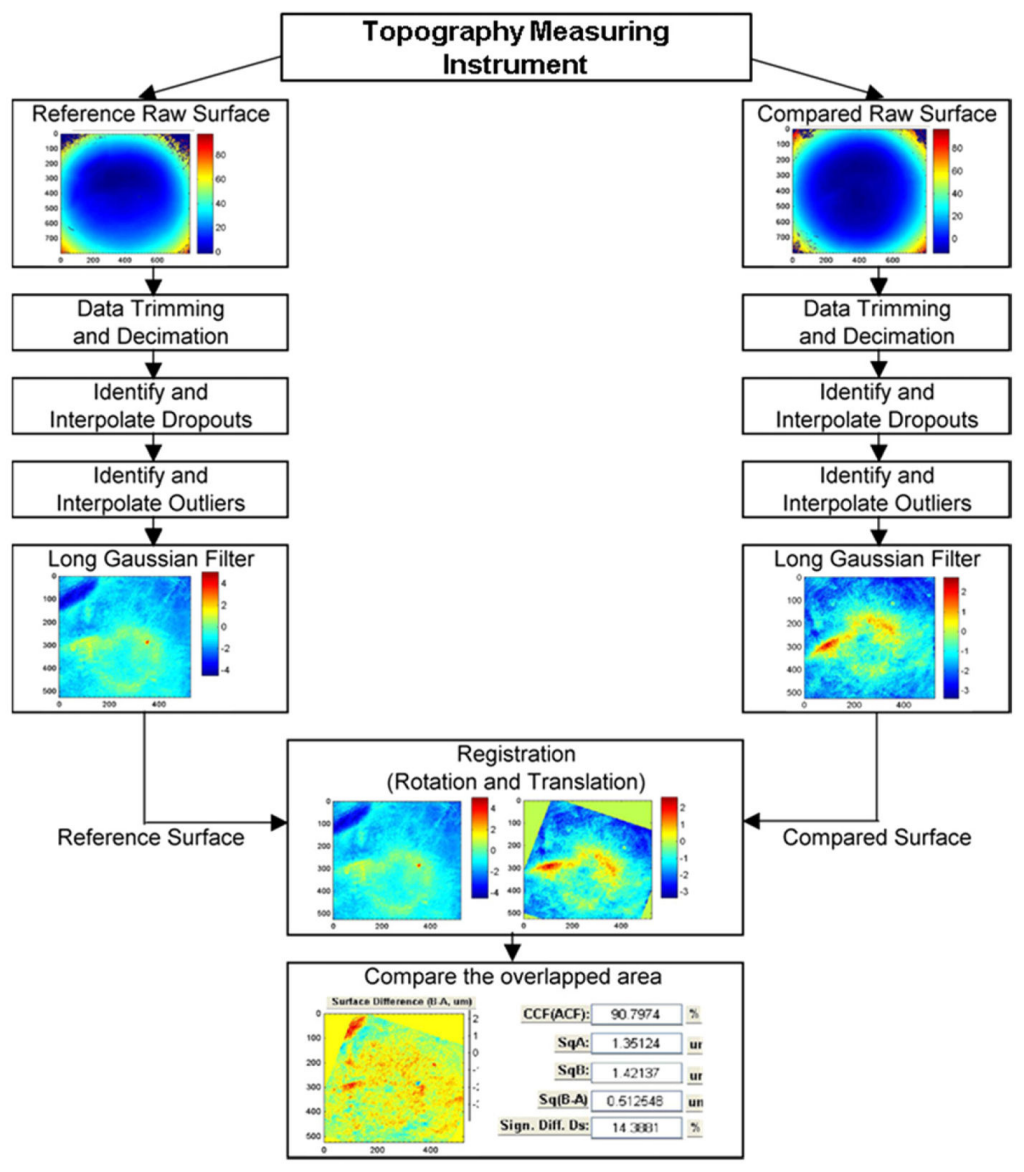
Illustration [16] of a procedure for assessing the similarity of two topography images: dropout and outlier detection, filtering, registration, analysis and parameters. In addition to the long scale filtering operation (shown) a short scale or smoothing filter may also be applied (not shown).

An example of a procedure for comparing two topographies is shown schematically in figure 18 [16]. Decimation may be performed to reduce the number of data points, for example, to speed the calculation. Bad data in the form of dropouts and outliers must be recognized and ignored or minimized. Then filtering is often performed to emphasize the individual characteristics [2], with sizes usually in the range of several micrometers to submillimeters, and to minimize long scale form features and unwanted short scale features, such as noise. Afterwards, the two surfaces need to be registered to assess whether similar features on them truly match up spatially. Finally, parameters quantifying similarity are obtained by various analytical methods. In the following sections, we emphasize the filtering stage and several different analysis stages.

### 3.2. Filtering techniques in ballistic identifications

Filtering is a standard procedure in surface metrology. Surface profiles (2D) and topographies (3D) normally include a wide range of surface spatial wavelengths, ranging from form deviation at long scales through surface waviness at mid scales to surface roughness at fine scales. For many topography applications and measurements, only a limited wavelength range, such as surface roughness, is of interest. An unambiguous extraction of the surface roughness from waviness and form deviation plays a key role in topography comparisons and measurements. Hence, filters are commonly used in surface metrology [44–48] and notably for the study of ballistic surfaces.

**Figure 19:**
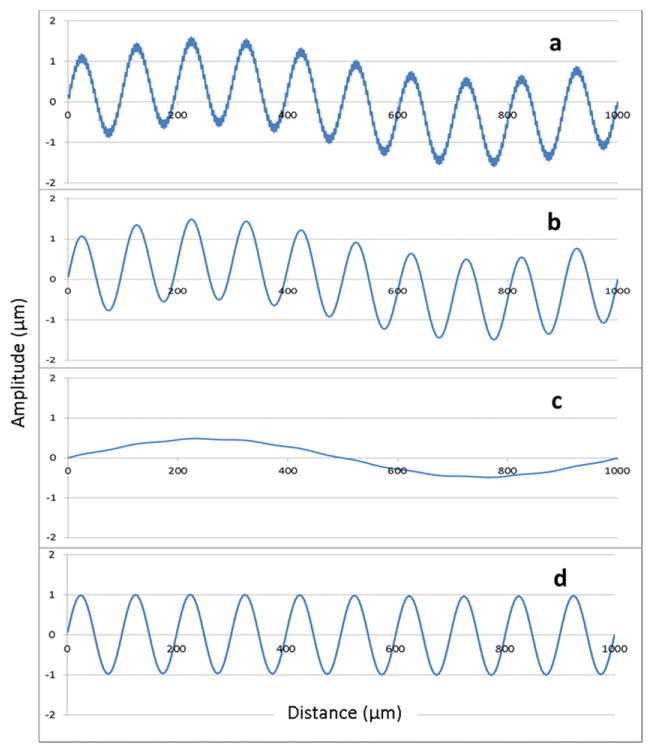
Illustration of a bandpass Gaussian filter; (a) segment of original profile with three sinusoidal components; (b) 25 *μ*m short wavelength filter attenuates the noise component; (c) 250 *μ*m short wavelength filter attenuates the roughness component; (d) subtracting c from b emphasizes the roughness component and attenuates the waviness component.

The most fundamental approach used currently is the digital Gaussian filter [44, 49]. This is a kind of moving-average, smoothing filter, where the moving average window uses a Gaussian weighting function. The smoothed profile that results can be subtracted from the original profile to produce a profile where the long wavelength features are diminished. The scale of features that are diminished or eliminated is given by the long cutoff wavelength (also called *nesting index* in a more general description) [45]. Conversely, if short wavelength noise is a source of confusion, the Gaussian smoothing filter may be applied with a short cutoff wavelength. Combining these two processes gives us a desired Gaussian bandpass filter defined by long and short cutoffs. Figure 19 illustrates how a filtered profile might appear. Figure 19(a) shows a segment of a longer profile containing the sum of three sinusoidal components: a waviness component with a wavelength of 1000 *μ*m, a roughness component with a wavelength of 100 *μ*m and a noise component with a wavelength of 4 *μ*m. We wish to emphasize the 100 *μ*m roughness component and attenuate the other two. Applying a Gaussian filter with a short wavelength cutoff of 25 *μ*m attenuates the noise component by about 94% while leaving the roughness and waviness components attenuated by less than 0.5% (figure 19(b)). Applying a second Gaussian filter with a short wavelength cutoff of 250 *μ*m attenuates the roughness component by about 98.7% but attenuates the waviness component by only 4.2% (figure 19(c)). Subtracting figure 19(c) from figure 19(b) reverses things and yields a relatively unattenuated roughness component while severely attenuating the waviness component (figure 19(d)).

An important limitation of the basic Gaussian filter is the loss of information at the ends of the original profile due to the moving-average windowing. Another limitation is the sensitivity of the filtered result to peaks and valleys in the data that which may not be of interest [50]. For these and other reasons, a wide number of other filtering methods have been developed and defined in documentary standards. These include Gaussian regression filters to address the end effects issue, robust Gaussian filters to minimize the sensitivity to data spikes, spline filters, morphological filters, and others. These exist in both 2D profile versions and 3D areal versions [51].

### 3.3. Standard surface topography parameters versus parameters that directly characterize differences of profiles and images

Surface topography measurements have played an important role for many types of industrial applications. Dozens of surface parameters are defined in national and international standards [26, 45, 52] and are specified on many types of product drawings. However, only a relatively few standard parameters are useful for quantitative comparison of surface topography features. Amplitude parameters, such as rms roughness, or spatial wavelength parameters, such as the mean spacing of peak irregularities, may be useful, by themselves or in combination, to relate to specific surface functions, but these do not provide enough information about detailed differences between two surface profiles or topographies. Not much information is obtained about similarity if we compare the rms roughness values of two surfaces. We need parameters that are sensitive to all the differences in detail between the features of one surface versus those of another. This is readily achieved with correlation [53, 54] and differencing methods [53, 55], but methods based around feature recognition [43] are also widely used.

### 3.4. Similarity parameters for topography measurements

During the development of NIST’s SRM bullets [23], Song *et al* used the cross-correlation function (CCF) to quantify the similarity of bullet signatures [56, 57]. The cross correlation function between two surface profiles *z*_A_(*x*) and *z*_B_(*x*) may be calculated by [53].

(1) $$\text{CCF}(A,B,\tau )=\lim_{L\to \infty }\;\left(\frac{1}{L}\int_{-L/2}^{L/2}z_{A}(x)z_{B}(x+\tau )\times dx\right)/\left[R_{q}(A)R_{q}(B)\right],$$

where *τ* is a shift distance between the profiles and *R_q_*(A) and *R_q_*(B) are the rms roughness values of the two profiles in the region of overlap. The cross correlation function for areal topographies may be calculated by [29].

(2) $$\mathrm{ACCF}=\left(\left(\sum_{m}\sum_{n}\left(A_{mn}-\bar{A}\right)(B_{mn}-\bar{B})\right)/\left({\left[\left(\sum_{m}\sum_{n}{\left(A_{mn}-\bar{A}\right)}^{2}\right)(\sum_{m}\sum_{n}{\left(B_{n}-\bar{B}\right)}^{2})\right]}^{1/2}\right)\right),$$

where the two arrays **A***_mn_* and **B***_mn_* here are the digitized surface topography images *z*_A_(*m*, *n*) and *z*_B_(*m*, *n*), and *m* and *n* represent indices in the *x* and *y* directions. Equation (2) is simply the discrete form of equation (1) extended to three dimensions. If two bullet signatures are identical, the value of the cross correlation function CCF_max_ is 100% at the optimum registration position. If two bullet signatures are similar but not identical—for example, two bullets fired from the same gun—their CCF curve has a correlation peak at the optimum registration position but with a CCF_max_ value less than 100%. On the other hand, if two bullets are fired from different guns, their signature patterns should have no correlation at all, and no significant correlation peak should be found on their CCF curve.

The CCF parameter is not a unique parameter for topography comparison because CCF is not sensitive to vertical scale differences. If two profile signatures A and B have exactly the same shape but different vertical scales, their CCF_max_ is still 100%. A parameter, called the signature difference, *D*_s_, is useful for quantifying both scale and shape differences between profile or topography signatures A and B [53]. It may be calculated as the normalized rms amplitude of the difference profile or difference topography image. For example,

(3) $$D_{s}={R_{q}}^{2}(B-A)/{R_{q}}^{2}(A),$$

where *R_q_*^2^ (A) is the mean square roughness of the reference signature *z*_A_ (X), used here as a comparison reference. When two compared profile signatures are exactly the same, *D*_s_ = 0. In this way, *D*_s_ is a complementary parameter to CCF.

Compared with existing proprietary algorithms and parameters used in commercial instruments for ballistics identifications, the proposed CCF_max_ and *D*_s_ parameters have several useful features:

- They are easy to understand and use; they are in the public domain, are amenable to open testing, and may be calculated from measurements traceable to the SI standard of length.
- The same basic parameters and algorithms can be used for quantifying signature differences for both 2D-profiles and 3D-topography images [57].
- If no scale differences exist between two signatures, there is a strong linear correlation between the parameters CCF_max_ and *D*_s_ [56]. In that case, either parameter can be used for representing signature differences for topography image comparisons and ballistics identifications.

**Figure 20:**
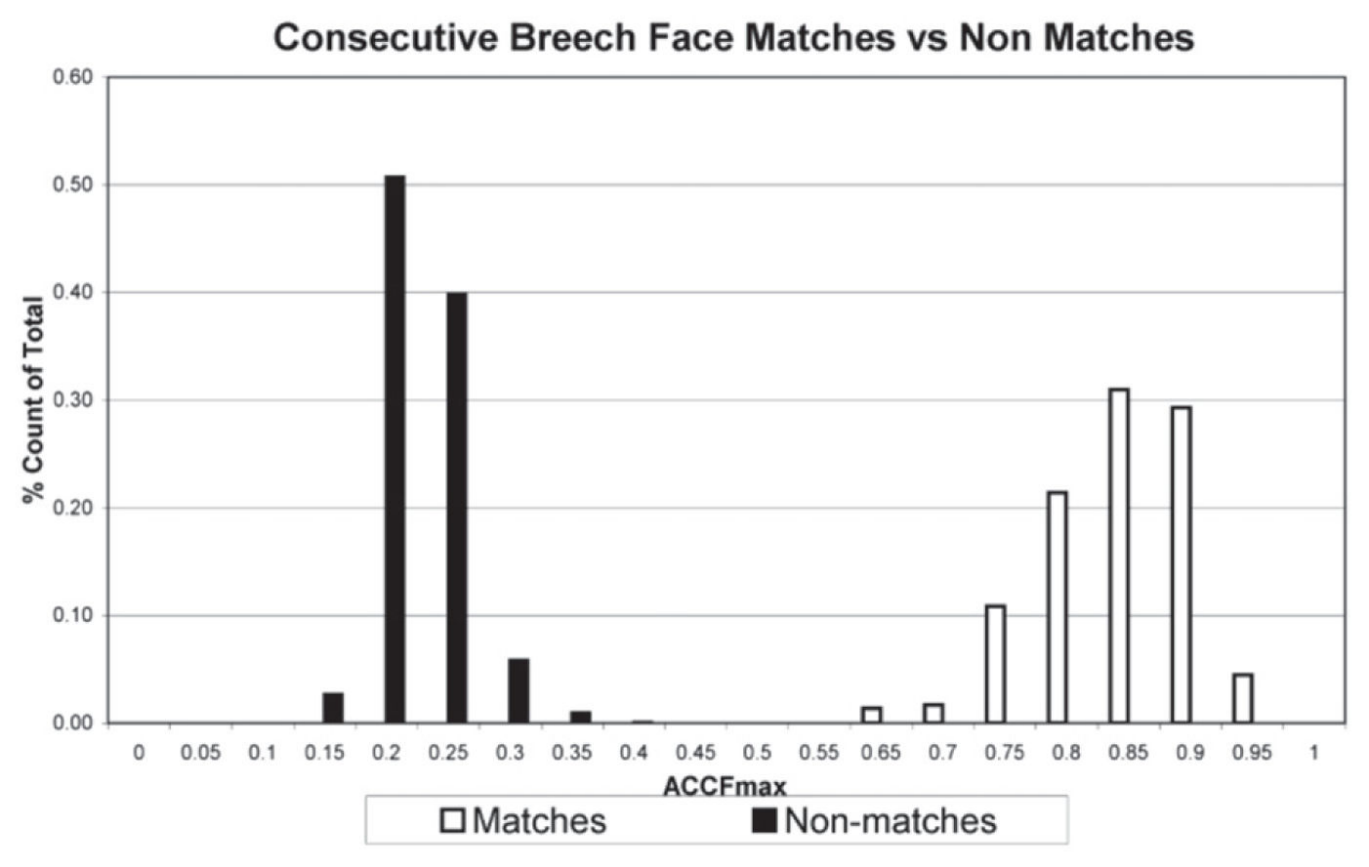
Data from Weller *et al* [58] showing cross-correlation comparisons using the CCF_max_ parameter among 90 test fires from ten consecutively manufactured breech faces. No overlap of data was observed between matching (same breech face) and nonmatching (different breech face) comparisons (©2012 American Academy of Forensic Sciences, reproduced with permission of John Wiley and Sons).

Weller *et al* [58] used the CCF_max_ parameter to compare topography images to identify spent cartridge cases from the same firearm slides. They started with ten 9 mm Luger caliber slides that were consecutively manufactured and that revealed both subclass characteristics and individual characteristics. This set of slides should be especially difficult to distinguish one from another. They obtained nine test fires from each slide, measured the topography of the breech face impression of all 90 cartridge cases, and performed cross-correlation calculations for the 8010 combinations of pairs. There were 7290 non-matching pairs, i.e., fired from different guns and 720 matching pairs. A graph of their results is shown in figure 20. Although this set of consecutively manufactured slides contained clear subclass characteristics, which could persist from one firearm to another, there is good separation between the cross-correlation values for the matching pairs and the non-matching pairs.

Another parameter, closely related to CCF_max_, which has been proposed for quantitative comparison is Chumbley *et al*’s ‘T1’ statistic [59]. Their method takes pairs of striated tool mark profiles and searches for a region of best agreement (as measured by a correlation coefficient) within a user-defined window. After this first step, referred to by the authors as ‘optimization’, a set of equally sized windows is chosen at random positions with respect to the region of best agreement. This set is paired with the comparable set of windows from a compared toolmark. The pattern of positions is the same for both profiles in the pair. If the pair of toolmarks is a true match, then one expects correlations between the randomly distributed segments to have high correlation values. Thus for matching tool marks, correlation of the distributed segments should produce *relatively high* correlation scores, and for nonmatching toolmarks the converse should be the case. The term, *relatively high* becomes quantitative when a second set of correlation values is calculated between segments which are now randomly chosen anywhere on the tool marks. If the two tool marks are a match, the correlation values from the first set of segments should be higher than the correlation values for the second set of segments. The comparison of the two sets of correlation values are ranked and transformed into Mann-Whitney U-statistics [60], yielding the T1 parameter. Distributions of the T1 parameter for known match and known non-match distributions can be used for hypothesis tests concerning unknowns.

**Figure 21:**
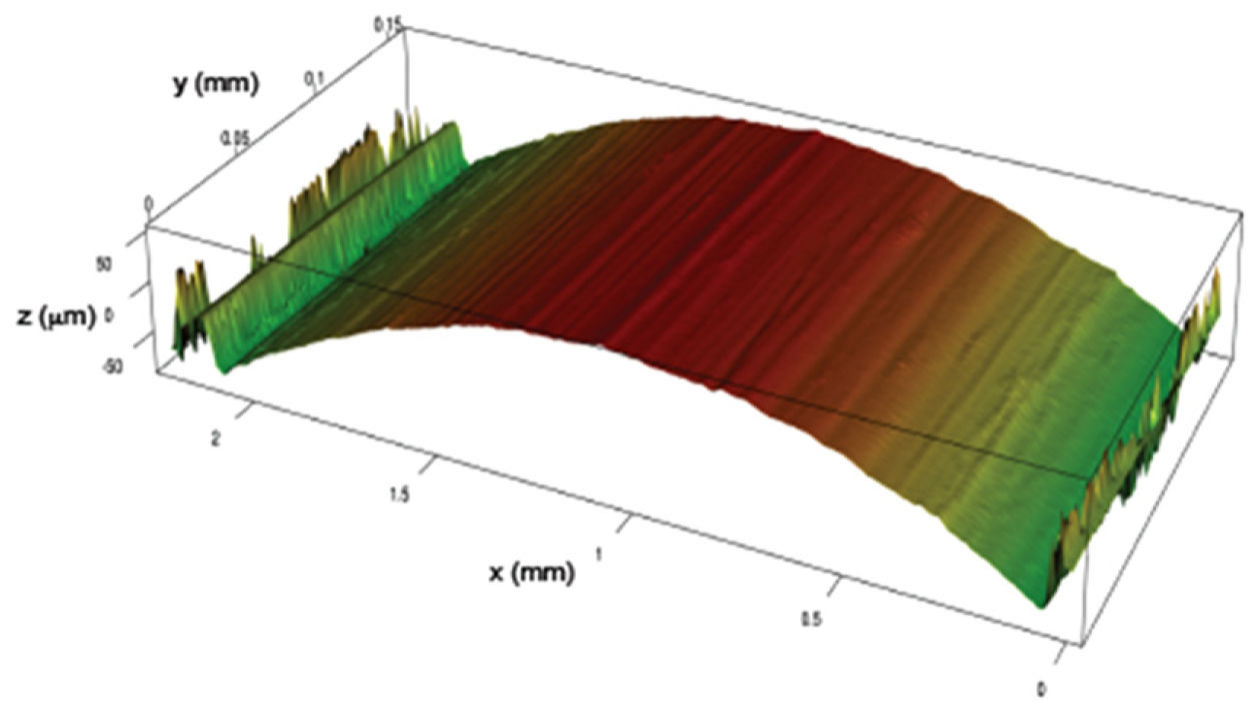
3D LEA from a 9 mm fired bullet acquired with a confocal microscope.

**Figure 22:**
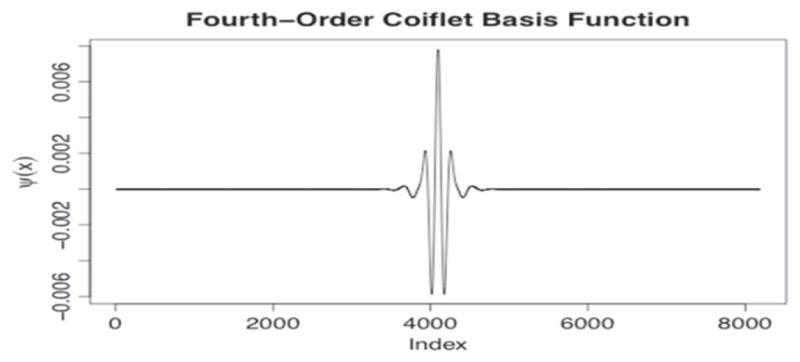
Fourth-order Coiflet wavelet basis function [65].

Various implementations of wavelet transforms have also been exploited for surface filtering and parameterization [61–63]. Figure 21 shows a 3D rendering of a land engraved area (LEA) on a 9 mm bullet taken with a Zeiss CSM-700 white-light confocal microscope at 50x. The striation lines are apparent but could be isolated and better defined before further analysis. For surface scale decomposition via wavelet transforms, Fu *et al* [64] have recommended the fourth order Coiflet basis (figure 22) because of its favorable band pass properties [65].

**Figure 23:**
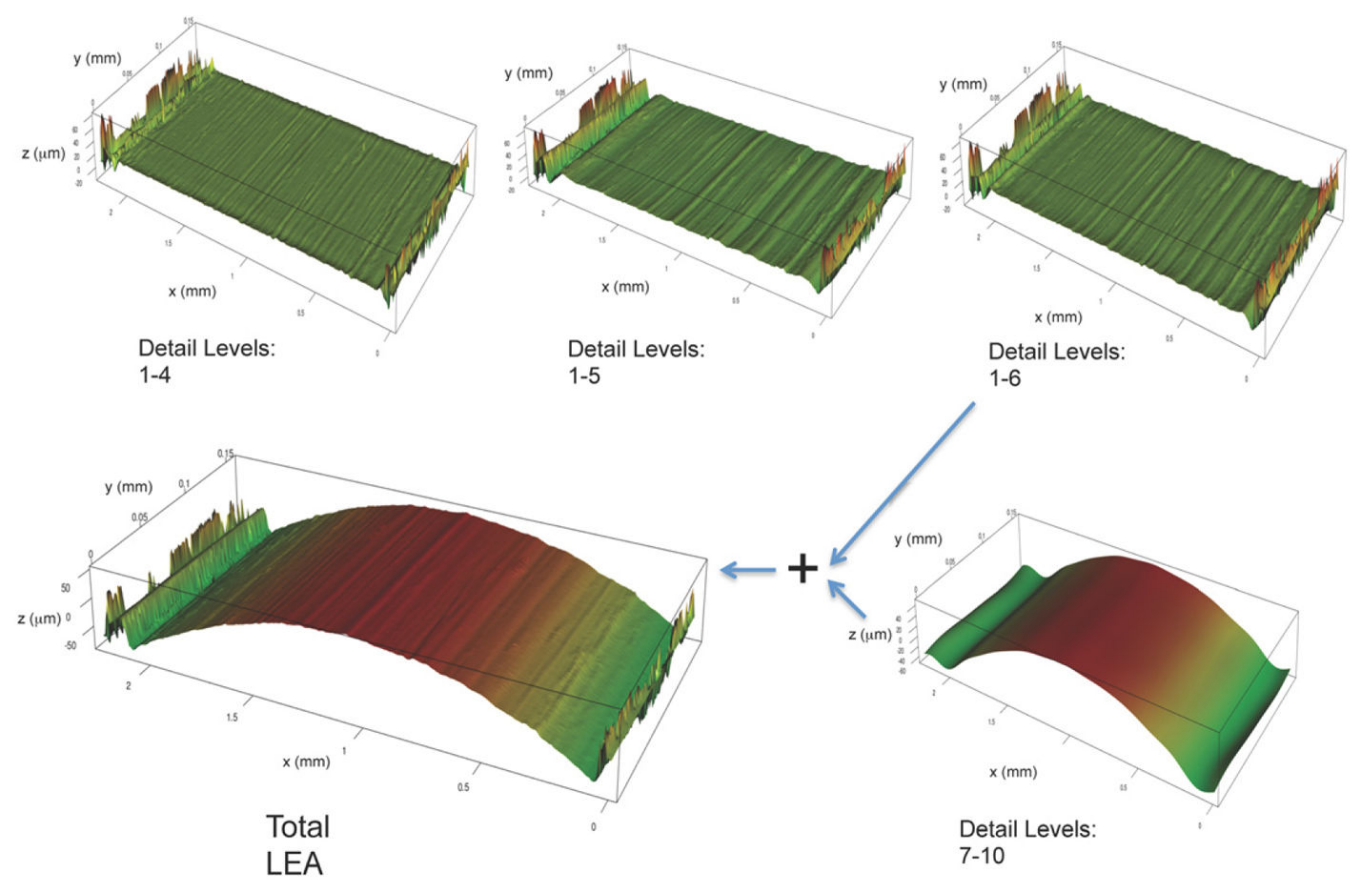
DWT decomposition of a LEA into detail (scale) levels.

**Figure 24:**
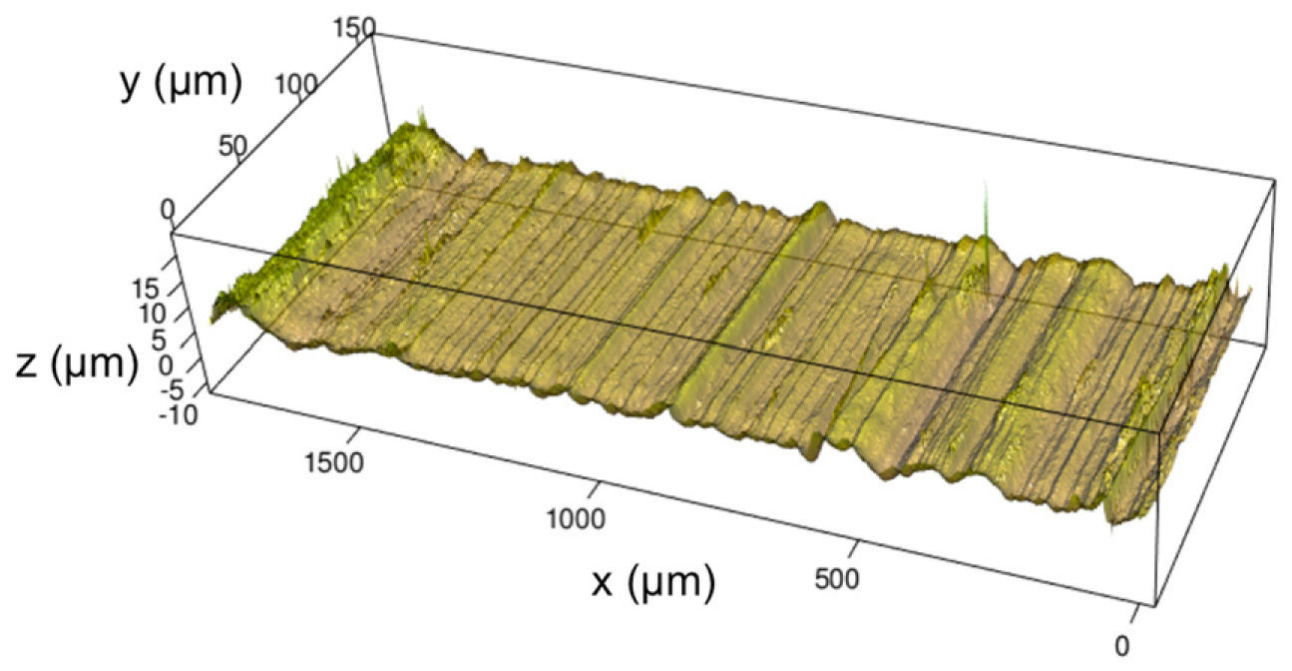
Striation line structure features extracted from the LEA of figure 21.

Using this basis combined with discrete wavelet transform (DWT) decomposition, the gun-unique striation structure can be made apparent. For example, the LEA of figure 21 appears again in figure 23. For this LEA, a combination of the wavelet detail levels 1 to 6 brings the gun-unique striation structure into vivid focus (figure 24) and filters out the long wavelength form.

### 3.5. Advanced statistical parameters

#### 3.5.1. Congruent matching cells (CMC)

Song has developed an analytical method that seems to improve on the basic approach of correlating entire images [66]. The method systematically divides measured 3D forensic images into ‘correlation cells’, and uses cell correlation instead of correlation of the entire images. This is done because a firearm often produces characteristic marks, or individual characteristics, on only a portion of the surface. If a quantitative measure of correlation is obtained from the entire areas of a pair of images, the correlation accuracy may be relatively low because some *invalid regions* may be included in the correlation [67, 68]. If instead, the correlation areas are divided into cells, the *valid regions* can be identified and the invalid regions can be eliminated. The use of a sufficiently large number of cells may provide a statistical foundation for estimating error rates from a well characterized population.

**Figure 25:**
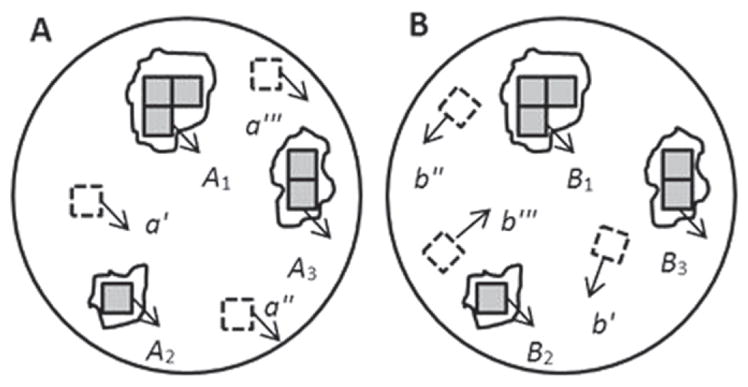
Schematic diagram of topographies A and B originating from the same firearm and registered at the position of maximum correlation. The six solid cells pairs are located in three valid correlated regions (A_1_, B_1_), (A_2_, B_2_), and (A_3_, B_3_). The three dotted cell pairs (a′, b′), (a″, b″), and (a‴, b‴) are located in the invalid correlation region.

The CMC method works as follows. If topographies A and B originating from the same firearm are registered at their position of maximum correlation (figure 25), the registered cell pairs located in their common valid correlation regions, as shown by the solid cell pairs located in (A_1_, B_1_), (A_2_, B_2_), and (A_3_, B_3_), are characterized by:

1. High pairwise topography similarity as quantified by a high value of the cross correlation function maximum CCF_max_;
2. Similar registration angles *θ*; and
3. Similar *x*–*y* spatial distribution pattern.

On the other hand, if the registered cell pairs are located in the invalid correlation regions of A and B, such as the dotted cells (a′, a″, a‴) and (b′, b″, b‴) in figure 25, or if they originate from different firearms, their maximum cross correlation value CCF_max_ would be relatively low, and their cell arrays would show significant differences in *x*–*y* distribution patterns and registration angles *θ*.

CMC pairs are therefore determined by four criteria, which must be satisfied simultaneously. The correlation value CCF_max_ must be larger than a chosen threshold *T*_CCF_ and the registration angle *θ* and *x*, *y* registration positions are within the chosen threshold limits *T_θ_*, *T_x_* and *T_y_*, respectively.

A fifth criterion is the number of matching cell pairs required to satisfy the above criteria in order to decide that two images are truly matching overall. Chu *et al*’s initial results for a set of breech face impressions [68] suggested that a pattern of six matching cells was a sufficient identification criterion for pairing up the breech face impressions that were studied. This parameter was called the CMC number and is similar to the concept of consecutive matching striae (CMS) developed by Biasotti and Murdock [69] for identification of bullet striation signatures. Thus, when the number of CMC pairs of the correlated topographies A and B is equal to or greater than C = 6, A and B are concluded to be a match.

**Figure 26:**
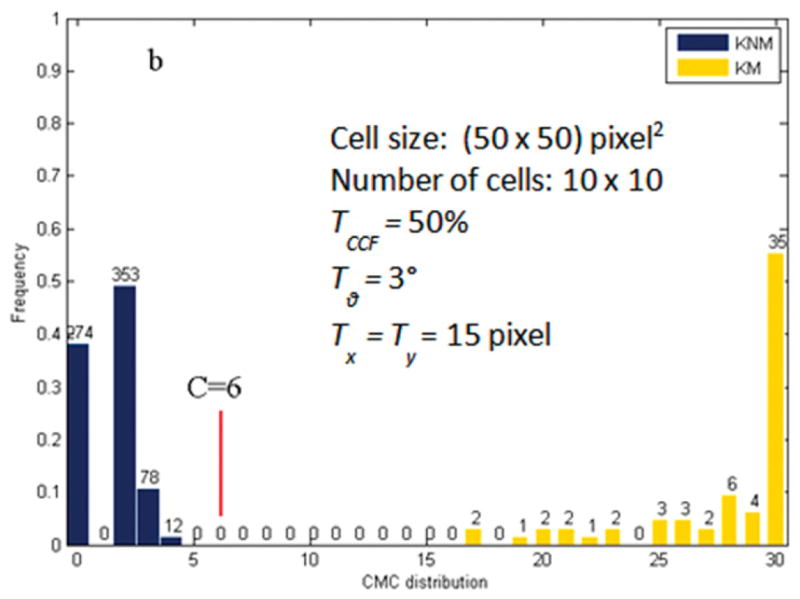
Relative frequency distribution (vertical axis) of CMC numbers (horizontal axis) for pairs of KM and KNM topography images (the KM and KNM distributions are each scaled to their particular sample size). For the 63 KM cartridge pairs, the CMC ranges from 17 to 30. For the 717 KNM cartridge pairs, the CMC ranges from 0 to 4. The KM and KNM distributions show significant separation without any false identifications or false exclusions.

**Figure 27:**
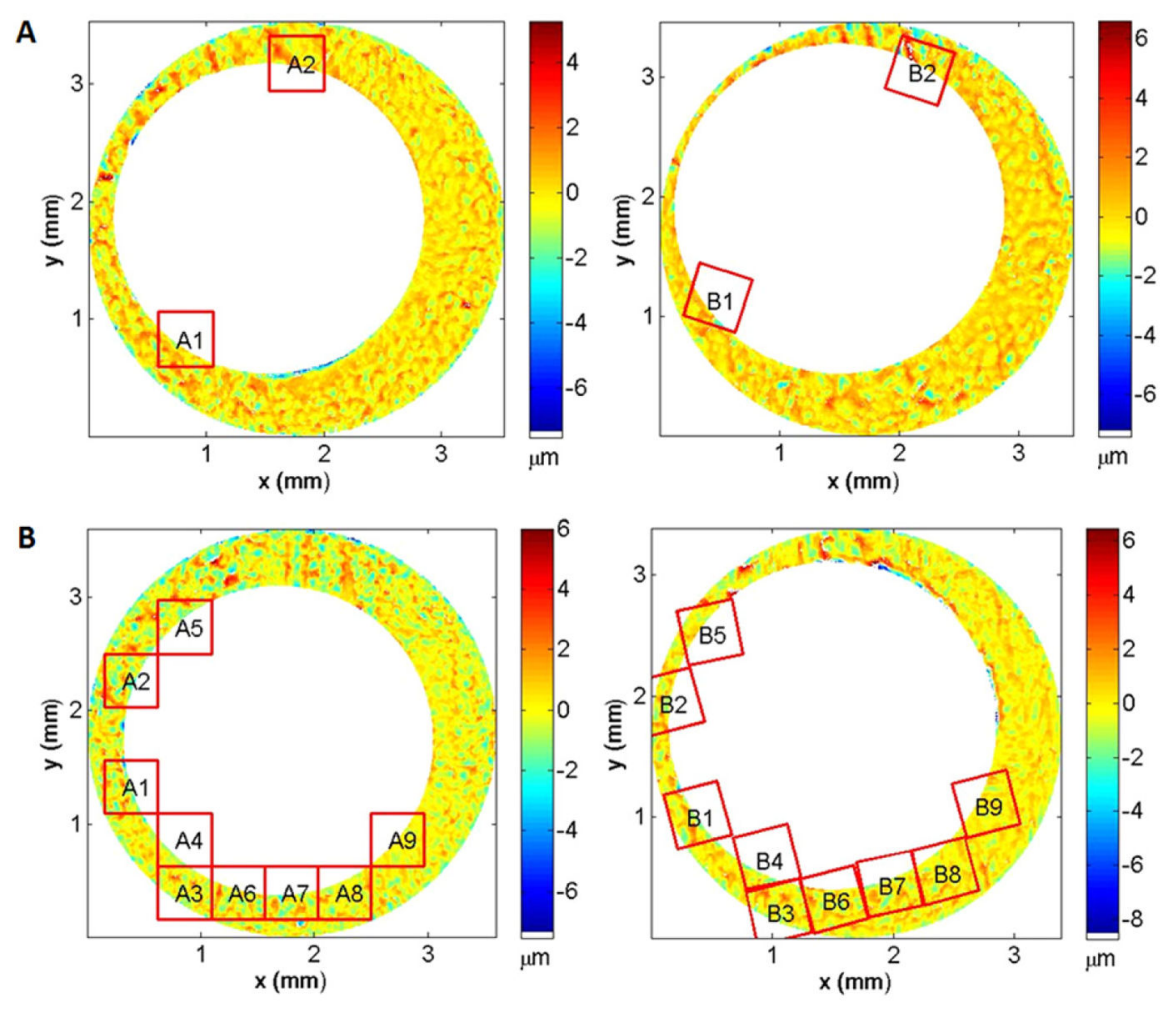
Depiction of correlated cells for two sets of correlated topography pairs. For the 717 KNM topography pairs, only five pairs had a CMC value as high as 2; one of these pairs of breech faces is shown in A. For the 63 KM topography pairs, only one had a CMC value as low as 9, this pair is shown in B. The pattern of cells A1–A9 on the left side of B is congruent within stated tolerances with the pattern of cells B1–B9 on the right. The surface topographies of the breech faces are depicted by the color scale of the diagram. The CMC method has also been adapted with success to matches and identifications involving conventional optical microscopy images [71].

Figure 26 shows Chu *et al*’s [68] results for the set of breech face impressions previously mentioned. A wide gap exists between the distribution of known matching (KM) pairs of impressions and known non-matching (KNM) pairs—a desirable result. Figure 27 from a more recent study by Song *et al* [70]—with the same data but with slightly different analysis parameters—shows correlated cells for two of the correlated topography pairs. For the 717 KNM topography pairs, only five pairs had a CMC value as high as 2 in that study; one of these pairs of breech faces is shown in figure 27(a). For the 63 KM topography pairs, one had a CMC value as low as 9; this pair is shown in figure 27(b). The pattern of cells A1 to A9 on the left of figure 27(b) is congruent with the pattern B1 to B9 on the right. The surface topographies of the breech faces are depicted by the color scale of the diagram.

#### 3.5.2. Principal component analysis (PCA)

**Figure 28:**
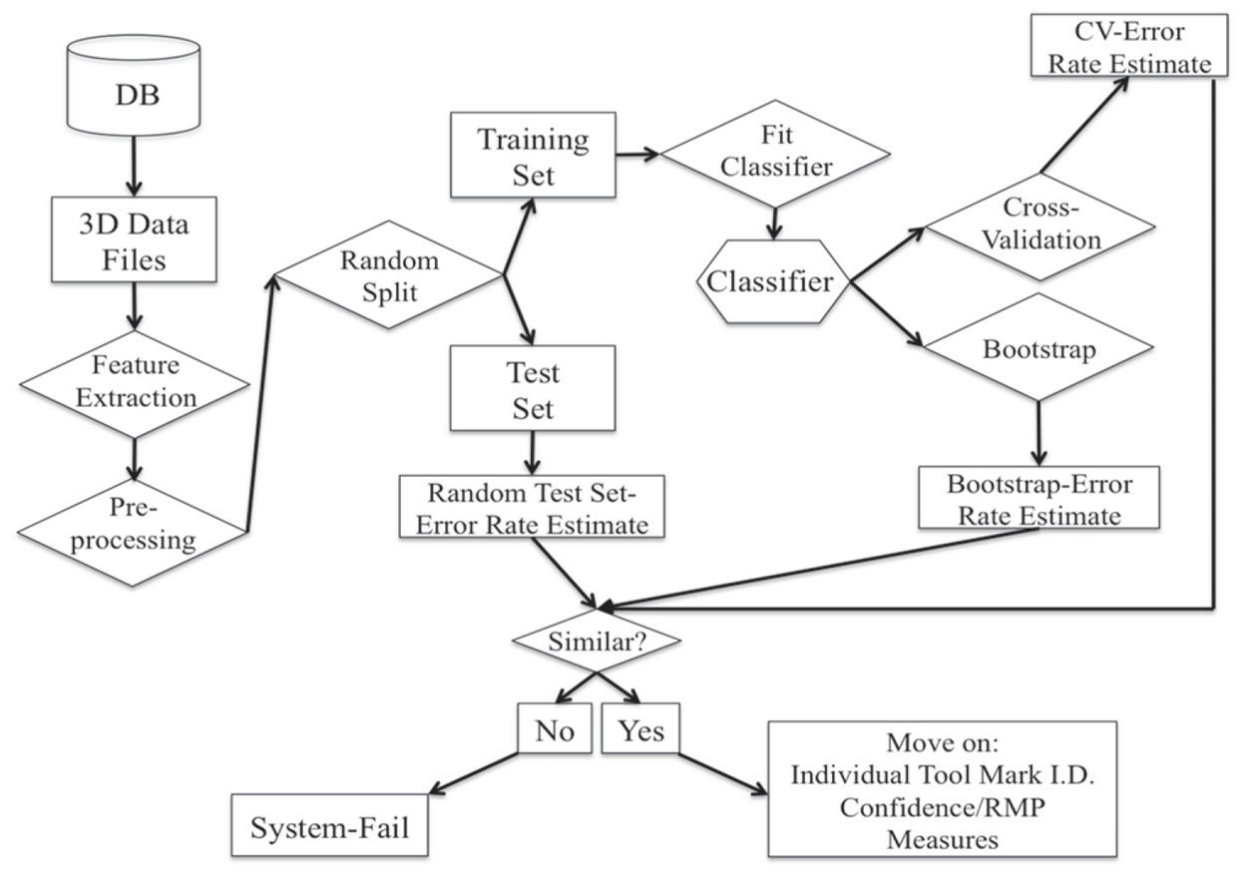
Machine learning work-flow for the identification of tool marks with 3D surface data.

An alternative to the cross-correlation approach to surface comparisons is the multivariate *machine learning* scheme discussed by Petraco *et al* [62, 63, 72]. A tool mark surface contains a tremendous amount of information. Most of the information is lost in summarizing the surface with a single number (i.e. a single univariate similarity metric). Instead, the machine learning approach derives a set of values to characterize surfaces. These vectors of *features* can be standard surface parameters [26, 52] or any other numerical or categorical values that potentially discriminate one surface from another, assuming that the surfaces are generated from different sources. The work-flow for the machine learning approach is laid out in figure 28.

A series of known tool marks, fabricated, for example, by the same type of tool, are recorded in 3D via known surface metrological measurement techniques [73]. These results are stored in a database. The surfaces are then pre-processed for analysis.

For the system constructed by Petraco *et al*, preprocessing first involves dropout/outlier interpolation. Next, feature extraction is performed to produce feature vectors of the surfaces. In the following, we will emphasize striation toolmark patterns produced with a ‘scraping’ action rather than impression patterns produced with the action of force applied perpendicular to the surface. Striation patterns require different feature sets in the analysis than impression patterns. Striation patterns are adequately summarized by mean profiles, which are often averaged along the surface in the direction of tool travel [22, 31, 59, 67, 74]. This is because the individual profiles on a striated surface are highly correlated, and striation pattern profiles may be represented with only a few kilobytes of information as opposed to several megabytes required to represent a 3D topography image. As databases become larger, the file size of surface images will become a major issue, and the data compression provided by a mean profile is useful. Petraco’s process therefore begins with the generation of mean profiles from the striation pattern surfaces obtained by averaging the profiles along the surface in the direction of tool travel as described above.

The surfaces are then filtered into roughness and waviness components via the methods and standards outlined in section 3.2. Feature vectors describing a data set must be of the same length. This necessitates an extra preprocessing step since each profile, even from the same tool, varies somewhat in length. Registration with a quick cross-correlation between pairs of profiles is performed to find translations that yield maximum, though not necessarily high, similarity (areas of overhang are padded with zeros) [62, 63, 75].

**Figure 29:**
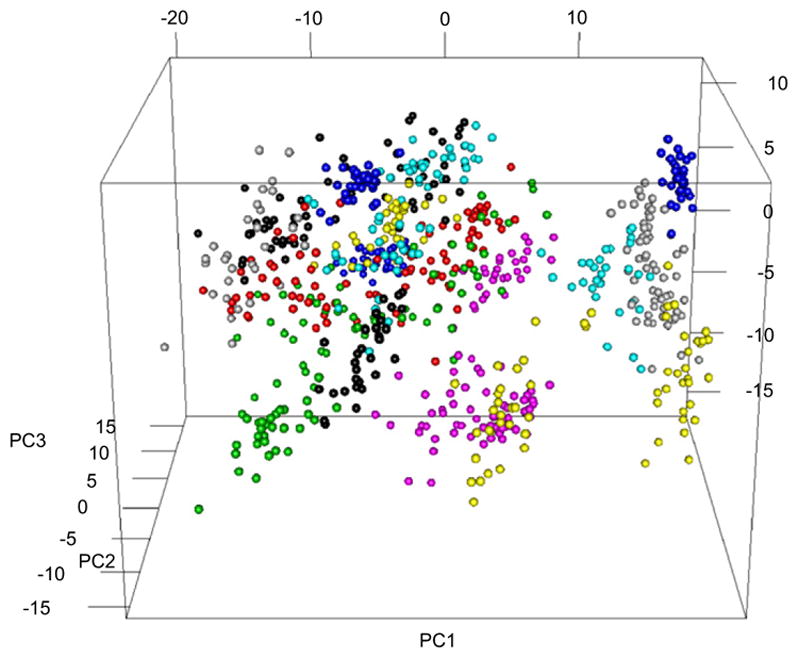
3D PCA of 760 real and simulated mean profiles of primer shears from 24 Glock handguns (newly drawn using data described by Petraco *et al* [28]).

Petraco then automatically extracts a set of features by applying PCA to a set of mean profiles. PCA effectively ‘compresses’ the tool mark profiles from many thousands of points to many tens of ‘effective’ points. Each ‘effective’ point is a linear combination of all the profile’s points. Each added feature accounts for successively decreasing amounts of variance [76]. The variance order of the new variables provides guidance for the reduction of the data’s dimensionality while retaining an adequate representation (see figure 31 below). For example, enough principal components (dimensions) are usually included in the analysis to account for 95% of the original variance in the data. Figure 29 shows 760 real and simulated striation pattern profiles from the primer shear produced by 24 different 9 mm Glock pistols. The figure shows the data projected into the space of only the first three principal components.

Each point in the plot represents a profile, and only three features of each profile can be illustrated in 3D. Therefore, for illustration purposes, each profile is shown as a point with three coordinates, whereas the number of features (principal components) could be much larger. The points are color coded as to which Glock fired the cartridge cases that provided the pro-file so there should be 24 color groupings in the chart. The 3D plot itself accounts for 45% of variance ‘information’ in the data set. Even with 55% of the variance thrown away, the 3D-PCA shows clearly that the tool marks are discriminable. Note however, we are representing each tool mark pattern as three numerical points. This is good from the standpoint that we can ‘see’ the discrimination between the tool marks, manifested by a visible clustering of tool marks made by the same tool. However, there is no *a priori* reason to expect three components (or one or two for that matter) to provide good discrimination. If four or more components are required, a visual assessment of discriminability becomes difficult if not impossible. Also, the value of using computational algorithms to discriminate tool marks is the capability to do *everything* numerically, including assessing discriminability.

Once a feature set is chosen and a matrix of feature vectors constructed, it is split randomly into training and testing sets. Machine learning algorithms are ‘trained’ to recognize tool marks in the training set with a high probability. The training is essentially a model fitting procedure with many methodologies to choose from. When a machine learning scheme is selected and fit, the discrimination functions are applied on the test set in order to estimate an overall error rate.

**Figure 30:**
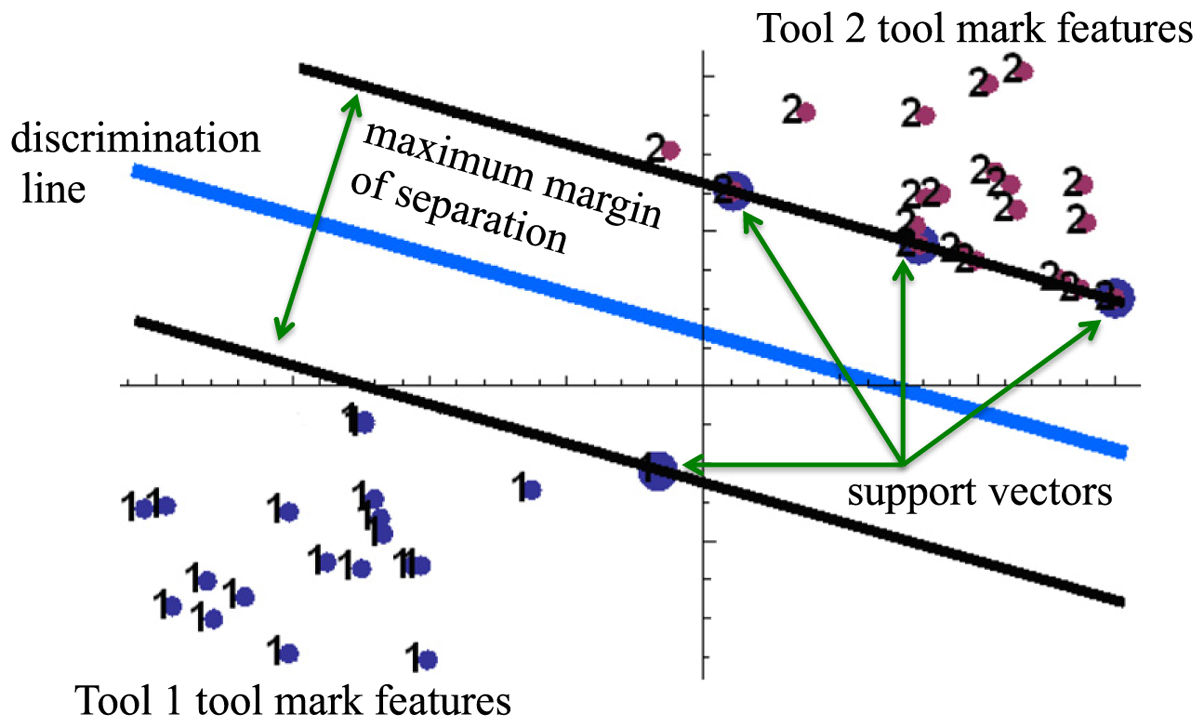
The job of the SVM algorithm is to determine the blue line, which separates the measurement data for tool 1 from tool 2. The large blue data points indicate tool mark features defining the blue line, i.e. the ‘support vectors’.

Choices must be made concerning the discrimination algorithm to be used and the method to assess intermediate error during the training/fitting process. Petraco *et al* [62, 63, 72, 75] have found that the support vector machine (SVM) discrimination algorithm combined with PCA and hold-one-out cross-validation (HOO-CV) (described in section 3.7.2) for model fit diagnostics to be a balanced machine learning scheme for forensic tool mark discrimination. SVMs look to determine efficient decision rules in the absence of any knowledge of probability densities by determining maximum margins of separation [77] between classes of objects (see figure 30). This procedure produces linear decision making rules for identification, while seeking large margins for error.

**Figure 31:**
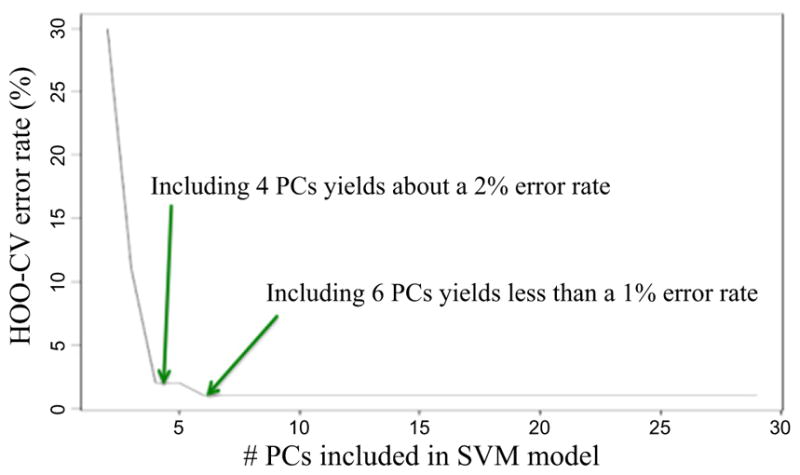
Typical HOO-CV error rate plot for SVM decision model fit.

SVMs are relatively easy to train, and tested/reviewed codes are available. The same is true for PCA [78]. HOO-CV is a standard way to decide on just how many PC-dimensions to use [78]. A HOO-CV error rate estimate is computed to assess SVM decision models with increasing number of dimension [79]. When a sufficiently low error rate has been achieved, that number of PCs is chosen as the model’s dimension. A typical HOO-CV error rate plot for the system designed by Petraco *et al* is shown in figure 31 [62, 63, 75].

Using the selected dimension, the model is bootstrapped (described in section 3.7.2) with the training set and tested with the test set. If the HOO-CV, bootstrap and test set error rates are similar and within the user’s predetermined tolerance, the tool mark identification system can then be used to identify true unknowns and assign confidence measures to the IDs made.

#### 3.5.3. Automated CMS

The method of counting CMS in optical micrographs to provide a criterion for identification of bullets was proposed by Biasotti in 1959 [80] and has been used internationally for bullet signature identifications since 1984 [69]. In 1997, Biasotti *et al* [81] refined the CMS criteria for identification to the following:

At least two different groups of at least three CMS appear in the same relative position, or one group of six CMS are in agreement in an evidence tool mark compared to a test tool mark.

**Figure 32:**
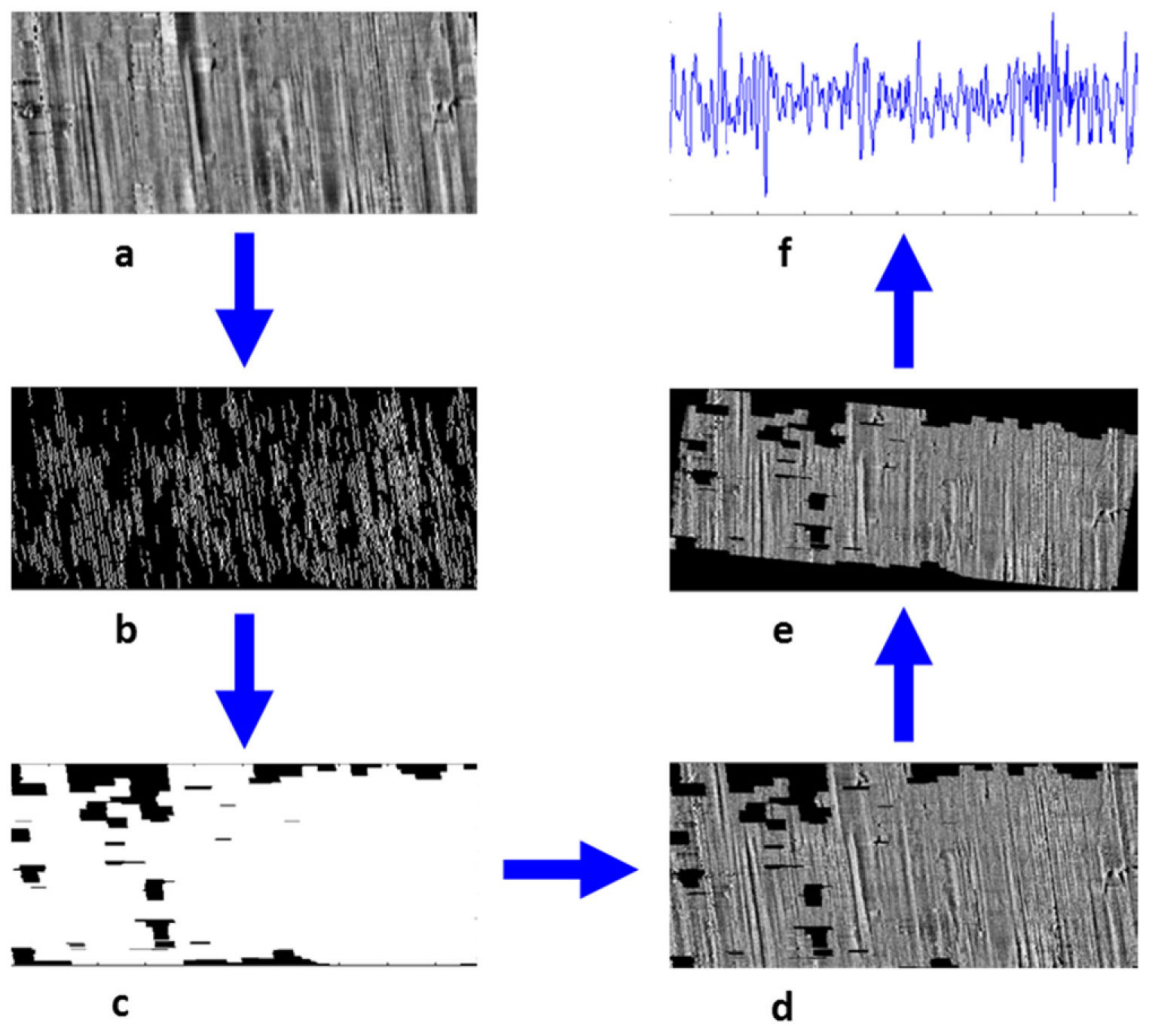
Preprocessing method of Chu *et al* results for bullets [83, 84]. (a) Preliminary processing, (b) edge detection and edge filtering, (c) mask image, (d) topography image with invalid areas removed, (e) rotated image of (d), (f) compressed profile.

The method has strong theoretical support [82], and recently Chu *et al* [83] adapted the method to analyzing topography images of bullets and developed automated criteria for recognizing areas of a bullet surface with valid striae, deep enough and long enough for use in the CMS evaluation process. Their preprocessing method [83, 84] is illustrated in figure 32. Figure 32(a) shows data acquired from a bullet LEA and filtered to minimize curvature. Figure 32(b) shows the significant striations as obtained with a Canny edge-detection method. The resulting areas that are masked for inclusion in further analysis are shown in (c) and the topography data with the mask applied in (d). These data are then tilted so that the striations are vertical as shown in (e) in order to calculate an average profile. The profile derived by averaging the data in (e) is shown in (f). Profiles like this one are analyzed for their similarities to one another using Chu *et al*’s automated simulation of the CMS method. With this method they were able to correctly match 14 out of 15 unknown bullets to one of ten consecutively manufactured barrels via a known set of ten pairs of bullets to which the unknown set could be compared. One result out of the 15 was inconclusive; there were no false matches.

### 3.6. Surface and profile simulators

An important step towards quantitative comparison of striation and impression tool marks is to understand the tool mark formation process and to assess the overall variability of tool marks from exemplar to exemplar. To date, three approaches have been implemented for forensic surface simulations and metrology applications.

**Figure 33:**
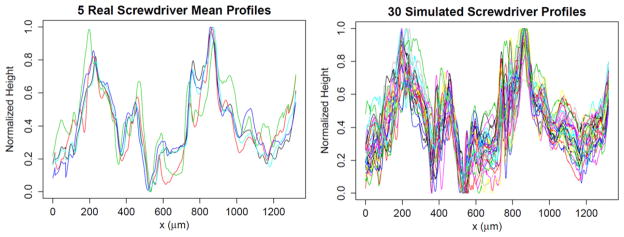
Thirty simulated profiles (right) based on five real profiles (left) from the same screwdriver via the simulation algorithm of Petraco *et al* [62].

The approach by Petraco *et al* [62, 63] provides stochastically generated virtual tool mark profiles that are simulated from actual tool mark (striation pattern) profiles fed into the system. Their idea is based on decomposing the tool mark into special scales in an objective way with a DWT. A profile is first decomposed into a set of *J* + 1 level coefficient vectors. Because the DWT is used, the length must be a power of 2 (i.e. profile length = 2*^J^* points, called *dyadic length*). Thus, padding or chopping to dyadic length is usually required. For each scale, vectors of wavelet-level coefficients from real profiles are collected together based on the tools they are known to have been produced by. Each column of the resultant ‘level-matrix’ represents how the coefficients vary for a local region across the real profiles (‘locality’ is dictated by the scale of the wavelet level). Non-parametric kernel density estimates (KDEs) are fit to the coefficients of each column. Samples of ‘simulated’ level coefficients are then drawn from the KDEs, level-by-level, and assembled into a ‘simulated’ wavelet transform. The inverse wavelet transform is then executed on the simulated sets of wavelet coefficients, yielding simulated profiles. An example of typical simulated profiles appears in figure 33 [62].

Bachrach *et al* [85] have recently reported on simulation software that also exploits wavelet decompositions of tool mark surfaces. Long wavelength shape and ‘brand’ (class) characteristics are extracted through the wavelet coefficients. However, unlike the system described by Petraco *et al*, the Bachrach software uses fractal analysis to include local ‘randomness’ components (i.e. fine scale surface roughness) into the simulated tool marks. This allows the random portions of the tool marks to be generated by predetermined parametric probability distributions. This contrasts with the Petraco system, which specifically uses empirical distributions. The Bachrach system is also capable of producing full 3D tool mark surfaces in a principled way by knitting profiles together with a first order auto-regressive [AR(1)] process.

Ekstrand *et al* [86] have developed a tool mark simulator that uses a model of a tool’s working surface, constructed from data obtained with 3D microscopy. Focus variation data were specifically reported, but the system can utilize data from any 3D microscopy. The geometry of the working surface is projected in the direction of tool travel. This identifies the highest points on the tool that scrape the deepest into the tool marking medium. A novel implementation scheme using graphical processing units (GPUs) was employed to significantly speed up the procedure. Notably the technique developed by this group can simulate tool marks produced by tools with arbitrary twist and angles of attack. Ekstrand *et al* plan to make their software available to the research community.

### 3.7. Error rate estimation

Reporting an error rate for firearm identification—that is, the probability that an identification is actually a false positive or the probability that an exclusion is actually a false negative—has been singled out as a fundamental challenge in forensic science [87, 88]. However, several methods have recently been developed to estimate error rates. These are described below.

#### 3.7.1. Automatic comparison of cartridge cases by Riva and Champod

**Figure 34:**
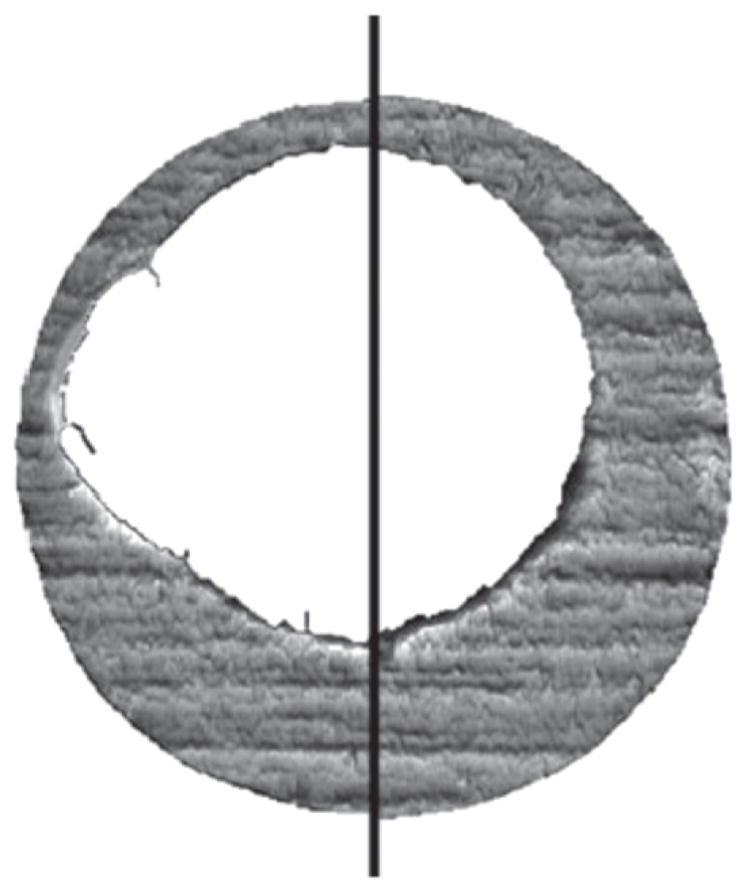
Side by side comparison of two breech face impressions from the same Sig Sauer P226 9 mm Luger firearm obtained with confocal microscopy by Riva and Champod (©2014 American Academy of Forensic Sciences, reproduced with permission of John Wiley and Sons).

Riva and Champod [89] developed an automated system for determining common-source identifications among a set of cartridge cases and providing error rates. They used a Nanofocus *μ*scan confocal microscope to measure the topography of the breech face impression and the firing pin impression of 199 cartridge cases. The data set consisted of one image each from 60 cartridge cases fired from a single gun, a Sig Sauer Model P226 9 mm Luger, one image each from 60 cartridge cases fired from a second gun, a Sig Sauer Model P228, and one image each from 79 other guns sampled from the Sig Sauer P226, P228, and Sig Pro Models. Correlations of pairs among the first set of 60 and second set of 60 provided two distributions of known matches (KMs), correlations among the third set of 79 provided a distribution of known non-matches (KNMs). Figure 34 shows a side by side comparison of a pair of topography images of the breech face impressions on cartridge cases fired from the same gun.

**Figure 35:**
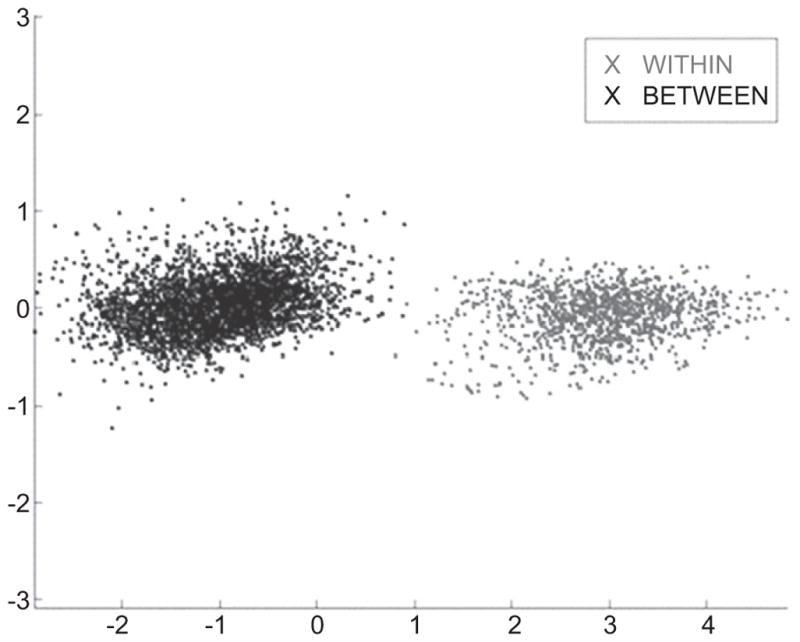
Distribution of data for known matching pairs (gray) of cartridge cases (all fired by one firearm) and known non matching pairs (black) versus the two principal components calculated by Riva and Champod [89] (©2014 American Academy of Forensic Sciences, reproduced with permission of John Wiley and Sons).

Separations between matching and non-matching pairs were achieved by PCA, and the resulting density distributions were found by KDE. Figure 35 shows an example of the separation achieved between the distribution of KMs for one of the guns and the distribution of KNMs, plotted versus the two principal components responsible for separation. Altogether Riva and Champod began with six distinguishing parameters, three each from the breech face and firing pin impressions. The clusters of points were fitted to probability density distributions (*p*), and likelihood ratios were calculated from those distributions. The likelihood ratio (LR) was given by

LR=p(R∣KM)/p(R∣KNM),

that is, the probability density for a result *R* among the KMs divided by the probability density for the result *R* among the KNMs. For one of their two examples, the separation between KM and KNM distributions was such that only 0.09% of the KNM results had LR > 1 and 0.26% of the KM results had LR < 1. In a hypothetical court room, a result corresponding to LR > 1 would help the prosecution case, and a result corresponding to LR < 1 would help the defense case.

#### 3.7.2. Multi-variate methods

**Figure 36:**
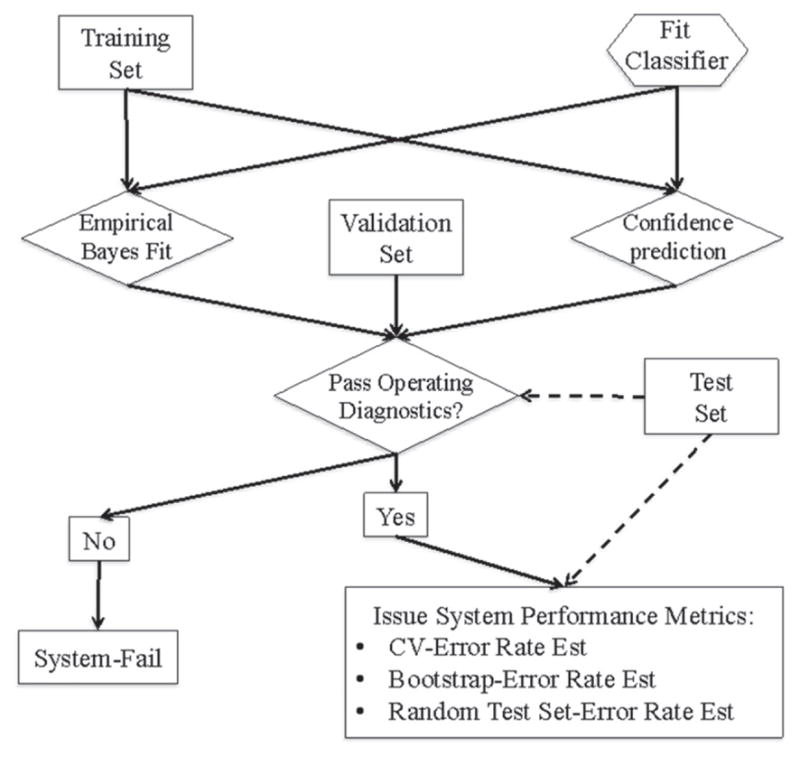
Error rate and confidence estimation schemes possible with machine learning approaches to tool mark identifications.

Exploitation of machine learning methods open up a myriad of approaches for error rate estimation. Figure 36 outlines a general work-flow.

Resubstitution is a straightforward method for empirically estimating an error rate with a machine learning technique [78, 79]. It involves applying the fit classifier to the set of data that was used to train it in order to produce the apparent error rate. The method provides a biased estimate, which tends to be overly optimistic and must be corrected. A standard improvement is the (refined) bootstrap [79]. A number of sets of bootstrap data (typically greater than 1000) are generated by randomly selecting (with replacement) *n* tool mark feature vectors from the original data set. Note that each bootstrap data set contains the same number of elements as the original data set. Thus, some patterns may be repeated. The classifier is retrained on each bootstrap set and an error rate determined on the original set. An average of the differences between the apparent and bootstrap error rates is found (a statistic known as the ‘optimism’) and added on as a correction to the apparent error rate to estimate a ‘refined’ bootstrap error rate. An advantage to this methodology is that it also gives approximate confidence intervals around the estimated error rate.

The HOO-CV [78, 79] method is an alternative to the bootstrapping procedure. This method fits the classifier using all but one of the tool mark patterns in the data set. The held-out pattern is then classified. A ‘1’ is recorded if it is misidentified, and ‘0’ otherwise. The hold-one-out procedure is repeated for each tool mark pattern in the data set, and the error vector is summed and scaled by the number of patterns to yield the HOO-CV error rate. A third technique is to put aside a large, though random, set of data to test the fit classifier after it has been trained [78]. It is prudent to make the test set be as large as feasible and the training set as small as feasible. Using these methods Petraco *et al* estimated error rates at the 1% to 5% level, depending on the size of the training data set, for distinguishing the individual sources for a set of shear mark impressions on Glock-fired cartridge case primer surfaces and a set of screwdriver toolmarks [62]. In both cases the data used were averaged profiles of the striated topography images. Ninety-five percent confidence limits on those estimated error rates were at approximately the 1% level, or smaller for large data sets.

#### 3.7.3. A feature based method

**Figure 37:**
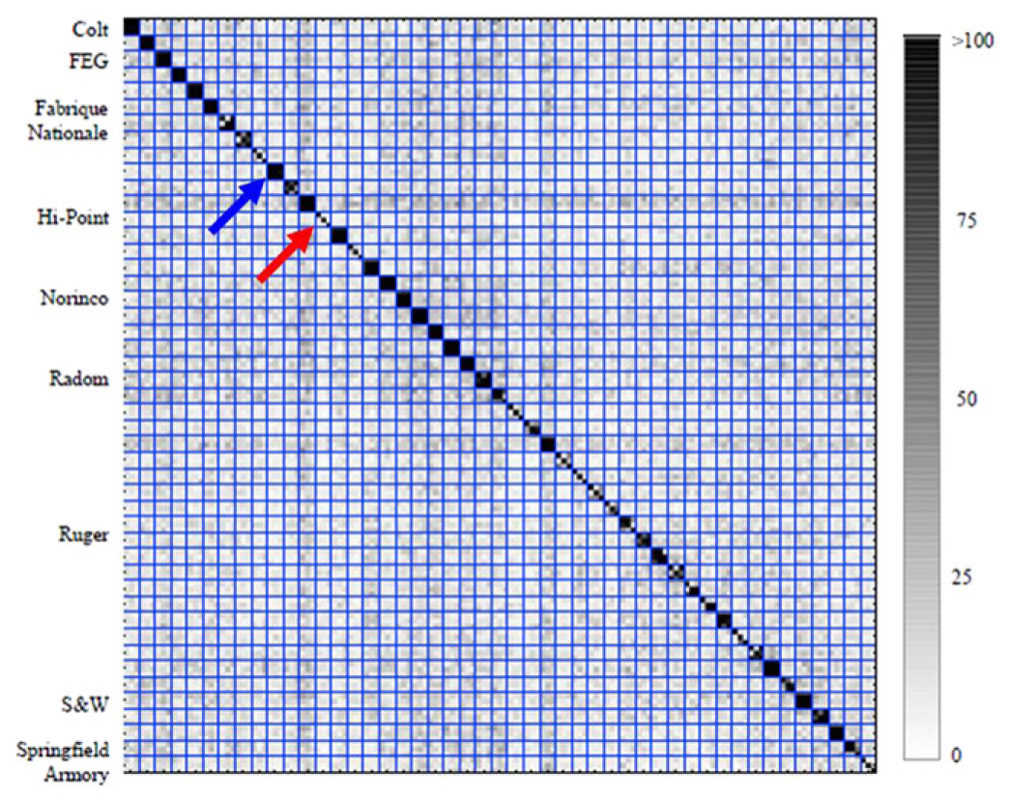
Results by Lilien of 19 981 comparisons among 141 cartridge cases (3 each from 47 firearms). ‘Each cell in the matrix corresponds to the match score between two casings (specified by the involved row and column)’. The firearms are separated in the matrix by blue lines. All cartridge cases fired by the same firearm are grouped into 3 × 3 cells along the main diagonal. The blue arrow (drawn by us) indicates an example where the separation of matches is well differentiated from non-matches. The red arrow (drawn by us) indicates an example where very little differentiation of matches from non-matches is occurring (originally published by the National Institute of Justice, US Department of Justice) [42].

Recently, Lilien completed a development study [42] of a commercial firearms identification system comprised of (1) a photometric stereo system with Gelsight imprinting for measuring the surface topography of breech face impressions and (2) a feature based system for characterizing the surface signatures and identifying matches. The system was tested in cooperation with the Oakland and San Francisco police departments. One of the tests involved 47 firearms of the 9 mm Luger type, and three test fires for each firearm. A round robin comparison of all test fires should produce 141 different matches among more than 19 000 possible combinations. Lilien’s software found 111 correct matches under criteria that the match score be greater than a certain threshold and should represent a ‘correct top result’. Notably, there were no false positives among the chosen matches. Lilien also developed a procedure to calculate a confidence level for these matches and claimed confidence levels of 99.99% or higher for 102 of the matches found. The details of these calculations were not yet published (however, see ‘note added in proof’ at the end). Figure 37 shows a ‘confusion matrix’ that plots the match scores as shades of gray for all comparisons. The overall array shows 141 × 141 comparisons. Cartridge cases fired by the same gun form close-knit 3 by 3 arrays straddling the central diagonal. Roughly 23 firearms stand out as highly identifiable, such as the one indicated by the blue arrow. Roughly nine fire-arms are much more difficult to identify, such as the one indicated by the red arrow, where the comparison of different cartridge cases from the same gun appear to give results that are indistinguishable from non-matches in this chart. Entries exactly along the diagonal are trivial cases where a single image is compared with itself.

## 4. Standards, traceability, and uncertainty for topography measurements

The Measurement Traceability Policy specified by American Society of Crime Laboratory Directors/Laboratory Accreditation Board (ASCLD/LAB-International) states that ‘The laboratory or calibration provider must document the measurement process or system used to demonstrate traceability and provide a description of the chain of comparisons/calibrations that were used to establish a connection to a particular stated reference’ [90]. In this section, we attempt to apply this directive specifically to topography measurements for firearms and tool mark identification.

According to the International vocabulary of metrology (VIM) [91], metrological traceability is defined as ‘property of a measurement result whereby the result can be related to a reference through a documented unbroken chain of calibrations, each contributing to the measurement uncertainty’.

In light of the above definition, three key steps for establishing metrological traceability and quality assurance for the topography measurements and imaging correlations of ballistics signatures have been proposed [92]:

- The establishment of reference standards for topography measurements,
- A chain of comparisons relating the reference standards to topography measurements of bullets, cartridge cases, and toolmarks, and
- The estimation of uncertainty in the measured quantities and/or the estimation of error rates in classifications and firearms identifications based on topography measurements.

We confine the discussion of these issues primarily to topography profiles and images. For conventional microscopy images, uncertainty and traceability have been discussed elsewhere in connection with image acquisitions and correlations performed within the National Integrated Ballistics Information Network (NIBIN) [12, 93] managed by the Bureau of Alcohol, Tobacco, Firearms, and Explosives (ATF). We cover each of the above topics in the subsections below.

### 4.1. Physical standards

Physical and documentary standards are critical for maintaining control in surface topography measurements. We discussed documentary standards earlier in connection with the discussion of measurement methods. The many types of physical standards for surface topography measurement are summarized elsewhere [94, 95]. In this subsection we focus on physical standards specifically for topography measurements and imaging.

**Figure 38:**
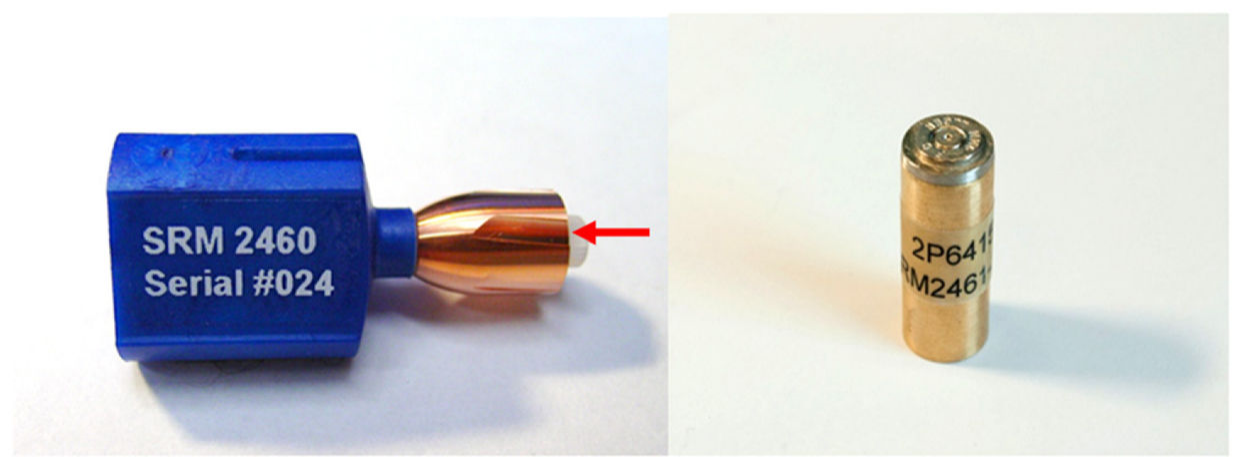
A SRM 2460 Standard Bullet (left) and a SRM 2461 Standard Cartridge Case (right). The red arrow indicates one of six land engraved areas around the periphery of the standard bullet.

Over the years crime laboratories have implemented quality control (QC) bullets and cartridge cases for testing the accuracy and reproducibility of their surface imaging systems. These are bullets and cartridge cases fired from a single firearm, kept in the central laboratory as a reference, which may be typical of firearms recovered during investigations. This firearm could be used successively over time to provide artifacts (QC bullets) for different laboratories or at different times. However, the QC bullets could have problems with uniformity and traceability. In the late 1990s, the ATF expressed the need for physical standards that would be more stable over time and more reproducible. In response, NIST developed SRM bullets and cartridge cases, SRMs 2460 [23, 56, 57] and 2461 [29], respectively (figure 38). These highly reproducible standards enable users of optical imaging and topography measuring systems to test the quality and stability of their systems from time to time and from one place to another.

**Figure 39:**
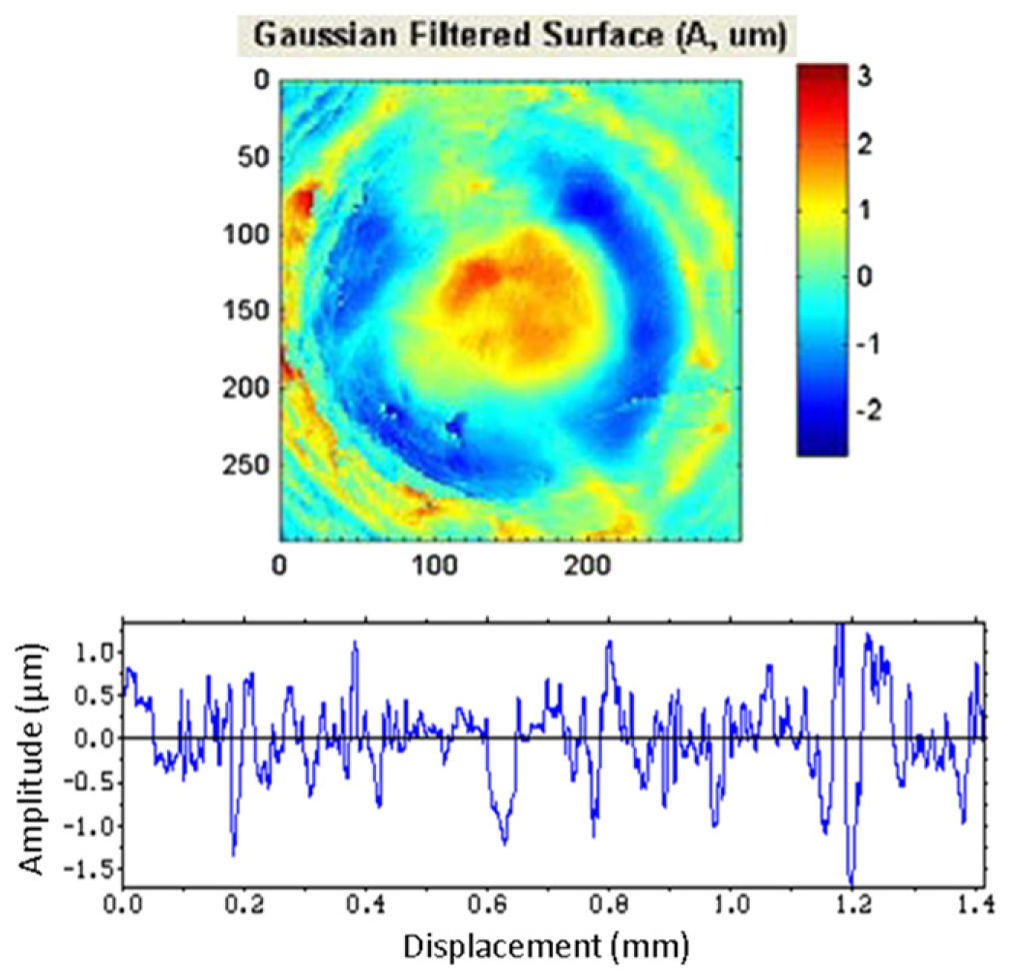
(Upper) topography image of the firing pin impression on unit 153 of SRM 2461, obtained by DSCM, filtered to remove curvature, and available online [97] as a master image. The units are in micrometer. (Lower) master surface profile for LEA 1 of SRM 2460, obtained by stylus profiling, filtered to remove curvature, and available on line [96].

**Figure 40:**
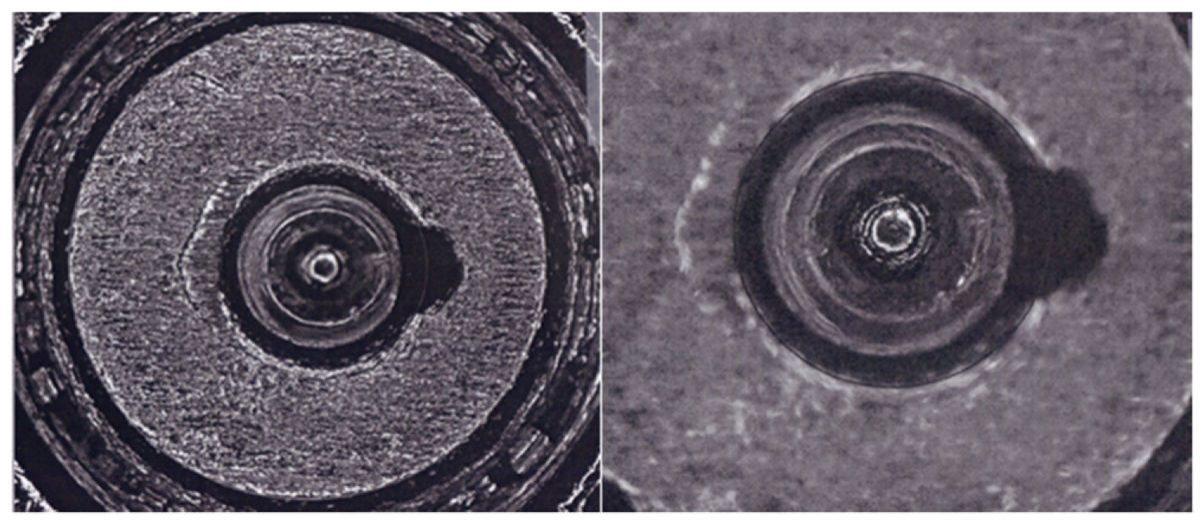
Golden images [93] of a unit of SRM 2461 acquired with IBIS BRASSTRAX and housed in a regional data base by the ATF National Laboratory, Ammendale, MD. A breech face impression is shown on the left. The field of view is roughly 4 mm by 4 mm. The firing pin impression is shown on the right and is roughly 1 mm in diameter.

For topography measuring systems, master profiles and topography images of the standard bullets and cartridge cases, respectively, are available online for downloading and correlation with users’ own topography measurements [23, 29, 96, 97]. Figure 39 shows two examples of data available online. For crime labs participating in the ATF’s NIBIN with IBIS optical imaging systems, the ATF maintains *Golden Images* of bullets and cartridge cases, acquired with IBIS workstations, to which NIBIN users can correlate [12, 93] their own acquired images. Figure 40 shows examples of these Golden Images. Users with other types of optical systems may develop their own Golden Images using the SRMs as well.

### 4.2. A chain of comparisons

**Figure 41:**
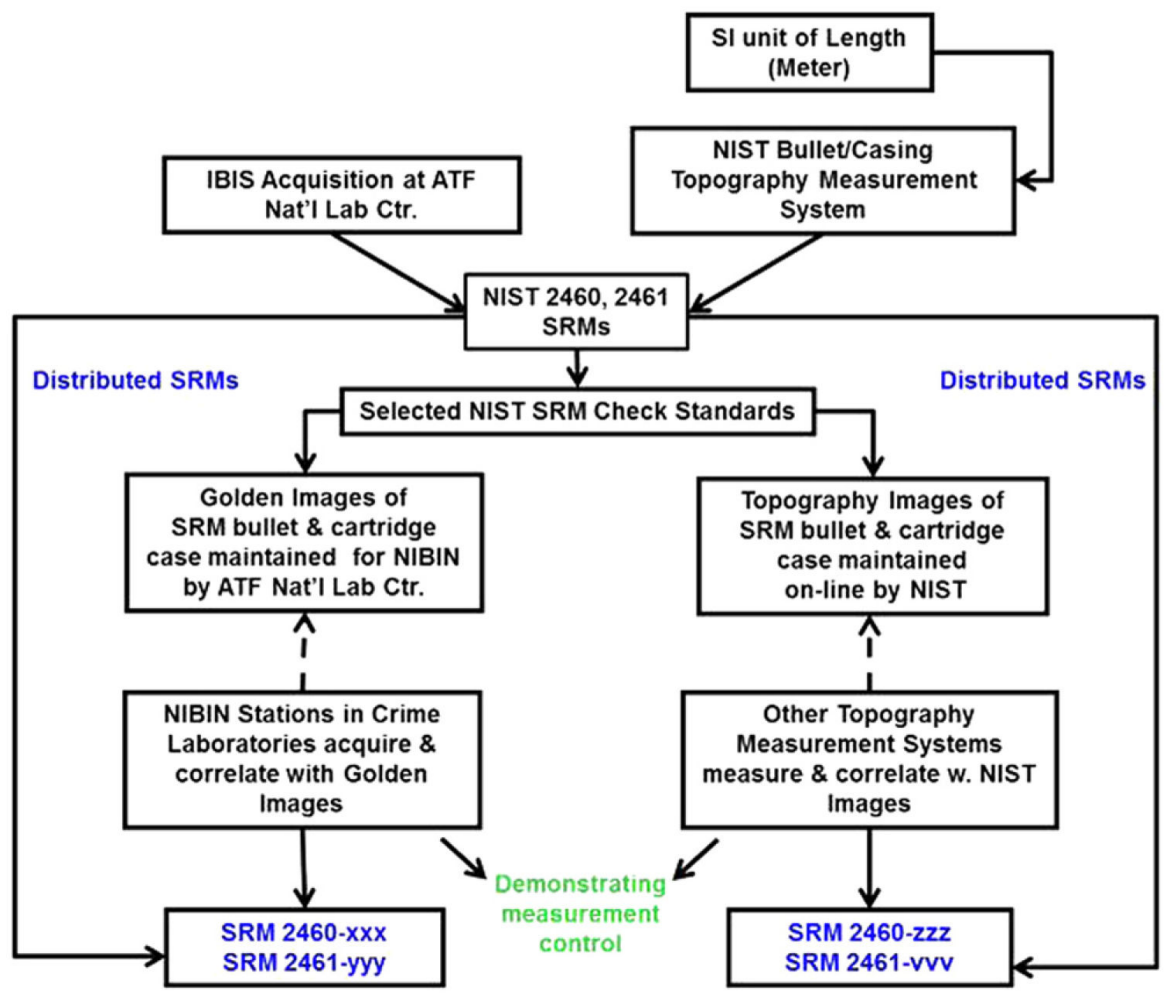
Establishment of a traceability and quality system for NIBIN acquisitions and correlations.

A flow diagram for the establishment of a Traceability and Quality System using the SRM bullets is shown in figure 41 [93]. For topography profiles and images, we emphasize the right side of the chart. The topographies of the SRM bullets are nearly identical to one another as are the topographies of the SRM cartridge cases. These similarities are quantified by the cross correlation maximum and the fractional difference parameters quoted on the SRM certificates of calibration [23, 29]. Most of the units of the SRMs are made available to industry, and a few are held at NIST as check standards for NIST’s own topography measurement QC. Since 2003, one of them, SRM 2460, Serial No. 001, has been routinely measured and correlated with a NIST master topography image more than 35 times and has demonstrated high measurement reproducibility: all the correlation values CCF_max_ are higher than 99% [12].

Topography images of the master surfaces are available online and may be downloaded for correlation. These include the profiles of all six LEAs of SRM 2460 Standard Bullet masters and master topography images of the breech face impression, firing pin impression, and ejector mark of the SRM 2461 Standard Cartridge Case. By correlating measurements of the user’s own SRM with the master profiles or images, the user can provide evidence that his/her topography measurements are accurate and that the user’s system can measure bullet and cartridge case surfaces similar to those of the SRM standard. Control charts can be used to further demonstrate that the system is stable over time [12, 93].

### 4.3. Uncertainty and error rate

The issue of uncertainty in topography measurements of bullets and cartridge cases largely amounts to the specific task of calculating an error rate for making identifications and exclusions about whether there is a common origin for a pair of surfaces using topography data and software analysis. The usual approaches to calculating uncertainties in the measured properties of a single object do not apply when two surfaces are compared for their similarity. Quantifiers of similarity between them need to be established as well as uncertainties in those quantifiers. For conventional, open parameters of similarity, such as cross correlation and relative difference [53, 56], the results are unitless and traceability to SI units is not relevant. Calculation of uncertainty and error rate for ballistic evaluations is still an evolving research issue.

We make the following observations about uncertainty using cross correlation as an example of a similarity metric. Sources of measurement error are likely to reduce the calculated cross correlation between two measured topography images, not increase it. If two topographies are measured by the same instrument, systematic sources of error are likely to cancel out. If so, they would not change the accuracy of the result. If they do not cancel out, the resulting errors in a series of correlations are likely to lead to variations in the results that can be recognized as statistical uncertainty. If two topographies are measured by different instruments and even more so by different methods, errors in either measurement lead again to reduced correlation values. Since errors of measurement generally lead to reduced correlation, we do not expect these errors to cause a decision error when a *positive identification* between two surfaces is made based on correlation results. However, if the correlation results suggest a choice of *exclusion* or *inconclusiveness*, the probability of error could be significant. Probabilities of error have been calculated from the statistical results from KM and non-matching surfaces for relatively small and controlled populations as discussed in section 3.7.

## 5. Other applications in firearm and tool mark identifications

### 5.1. Topography measurement and analysis of bullets

As part of an extensive study, Bachrach *et al* [98] investigated the surface topography of bullets fired by eight different brands of barrels of the same 9 mm caliber. A number of barrels of each brand were tested, each was fired 24 times, and two different types of ammunition were used. Then topography measurements were taken of the fired bullets using a confocal microscope. One of the striking results from that study was the observation of differences in the capability of the firearms to be identifiable and reproducible. Some brands had fine finished barrels with smooth surfaces and so produced weak tool marks. Other brands likely had looser tolerances on their barrels and produced tool marks that were not very reproducible from one shot to another. However, certain brands produced strong tool marks that were reproducible. Bachrach *et al*’s results lead to the conclusion that brands of firearms where the barrel finish is rough but the dimensional tolerances are tight will be better suited for firearm identification from fired bullets than barrels produced under other manufacturing conditions.

### 5.2. Topography of tool marks

**Figure 42:**
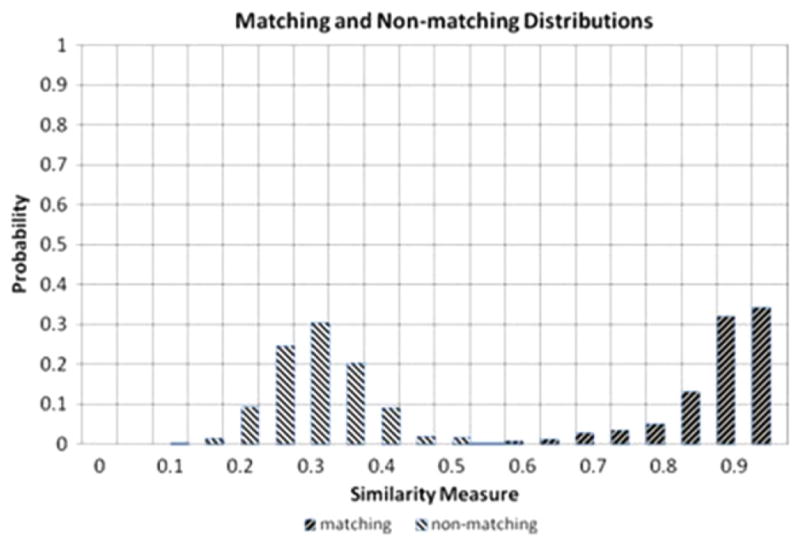
Matching and nonmatching distributions obtained by Bachrach *et al* [31] of similarity values for screwdriver striations on lead sheet at a tool angle of 30 degrees (© 2010 Intelligent Automation, Inc. Journal compilation © 2010 American Academy of Forensic Sciences, reproduced with permission of John Wiley and Sons).

Bachrach *et al* and Baiker *et al* measured the topography of striated tool marks produced by screwdrivers [31, 99], and by tongue-and-groove pliers [31] and performed systematic analysis of the differences in correlation among matching and non-matching pairs. Both groups found a high degree of separation between matching and non-matching pairs of tool marks from screwdrivers as long as they were produced under similar conditions, in particular, the same tool angle with respect to the surfaces. Figure 42 shows one of Bachrach *et al*’s graphs illustrating the separation that is achievable between the correlation distributions for matching and non-matching pairs.

Bachrach *et al* also formulated a straightforward parameter to characterize the overlap between matching and non-matching distributions, which they termed the *empirical error rate*. Characterizing the overlap (or inversely the separation) between correlation distributions is tantamount to quantifying error rates in correlations, that is, the rate of false identifications and false exclusions. A small overlap implies a large separation, which means small error rates and vice versa.

**Figure 43:**
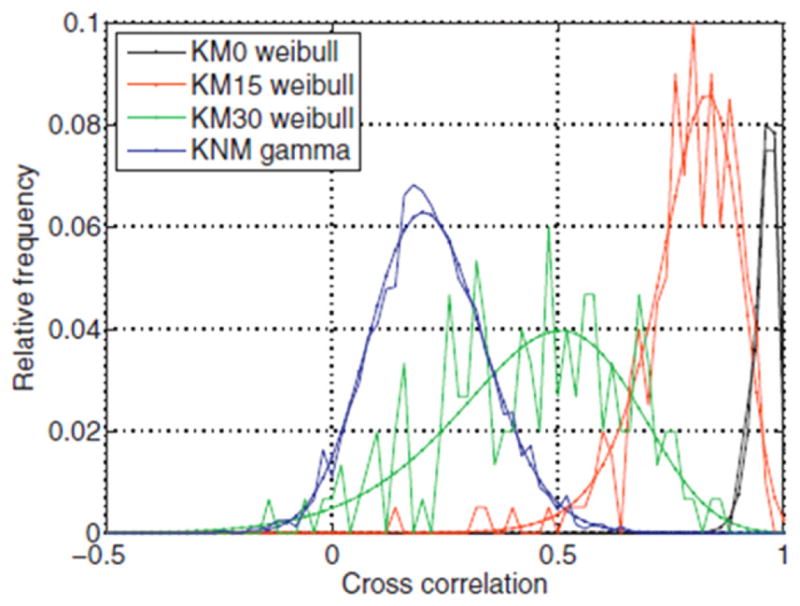
Distributions obtained by Baiker *et al* of correlation results for pairs of striated tool marks from screwdrivers. Far right (black)—known matches where the tool angle was the same for both tool marks, center right (red)—tool angles differed by 15°, center left (green)—tool angles differed by 30°, far left (blue)—different tools (known non-matches). Three sets of data were fitted to the Weibull distribution, one set to the gamma distribution (© 2014 reprinted with permission from Elsevier Ireland Ltd.) [99].

Both authors observed a degradation in the correlation between matching pairs when tool marks made at different angles were compared. Figure 43 shows a graph from Baiker *et al* [99] illustrating good separation between matching and non-matching distributions when the tool angle is the same for pairs of tool marks and a degradation in the separation when the angles of the tool are different. Bachrach *et al* also studied the effect of different substrate materials on the individuality of tool marks. Overall, the two studies lend strong support to the observation by Bachrach *et al* that ‘although it is not possible to prove uniqueness statistically, the results … provide support for the concept that tool marks contain measurable features that exhibit a high degree of individuality’ [31].

### 5.3. Alternative and supplementary topography parameters

Chu *et al* have performed several studies using confocal microscopy for topography measurement of cartridge cases and bullets and have developed a database for bullets [13]. Their work emphasizes other measurable quantities in addition to cross correlation functions for discerning individual characteristics and identifying bullets or cartridge cases fired by the same firearm. These other parameters include the parameter of CMS obtained from topography measurements of bullets and, by extension into 3D, the sizes of areas of correspondence between matching 3D objects, such as cartridge cases [100].

### 5.4. Commercial turnkey crime-lab systems

A number of commercial systems that measure topography and perform correlations are now available for use in crime labs. These include the IBIS TRAX HD3D from Ultra Electronics Forensic Technology Inc. [101, 102], the Evofinder from ScannBi Technology [103], ALIAS from Pyramidal Technologies [104], and the Topmatch-GS 3D system from Cadre Forensics [105].

## 6. Spinoffs in surface metrology

### 6.1. Comparing methods and instruments

Surface metrologists are often faced with the question: when two instruments measure the same surface, do they get the same result, and if not, why not? The answer might not only involve comparing surface parameters obtained with different instruments, but also direct comparisons of the profiles or topography images themselves. Profile and topography comparison is therefore a useful tool for instrument characterization [54]. Physical standards for surface roughness specified in national and international standards [26, 94, 95] can be used for this purpose, but we have also used a SRM standard bullet for profile comparisons between three optical instruments and a stylus instrument [106]. The high uniformity of the 2D bullet profile signatures along the lay of the SRM 2460 bullet made it possible for re-location of the bullet on different instruments to compare the ‘same’ profile.

**Figure 44:**
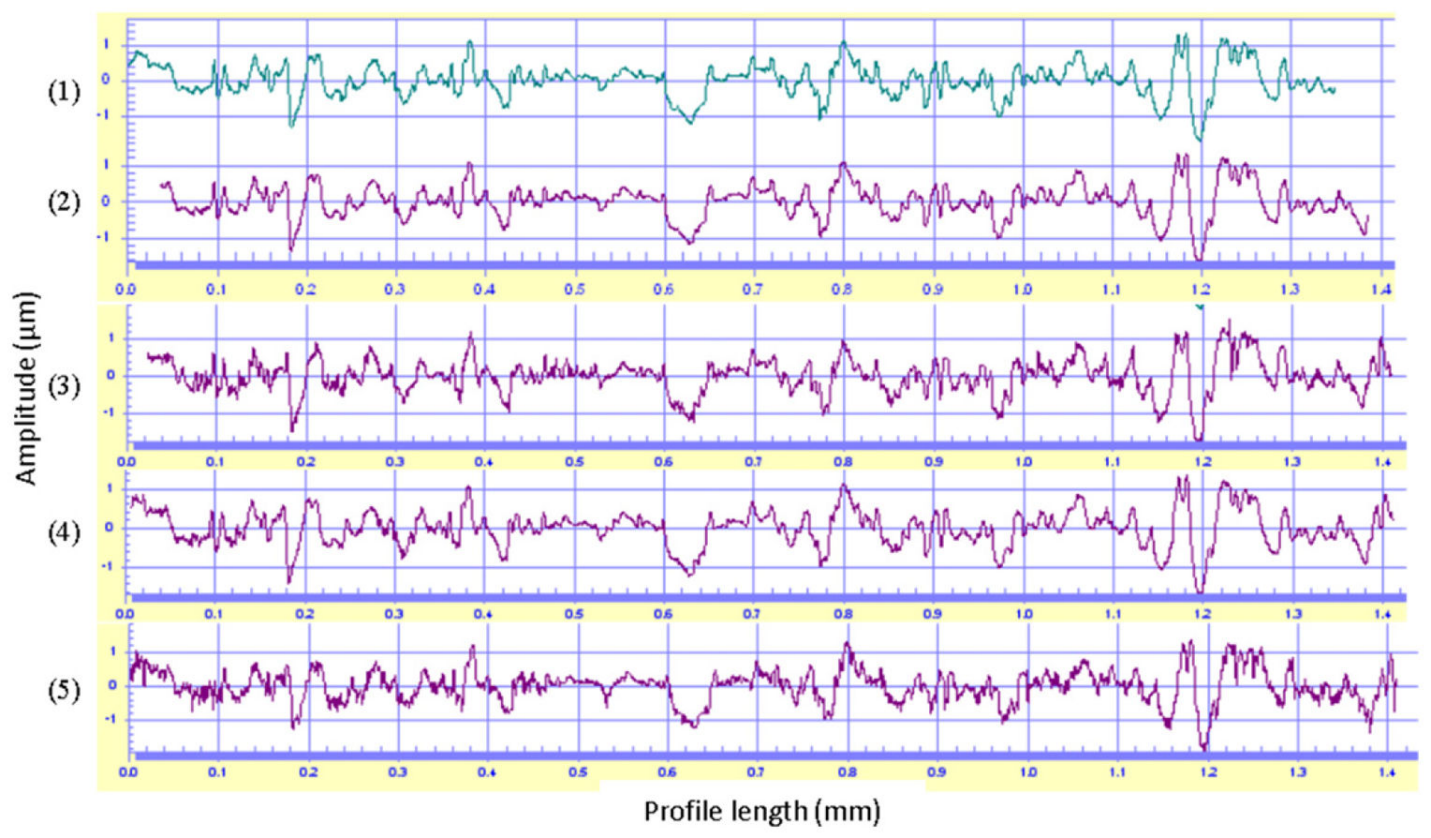
The profile of a land impression on a SRM standard bullet was measured by four techniques and compared with the profile of the virtual standard traced by a stylus instrument on the master bullet [106]. Profile (1) shows the virtual bullet profile signature standard. Profiles (2) to (5) show those measured on the same area of a SRM bullet by: (2) the same stylus instrument; (3) an interferometric microscope; (4) a disk scanning confocal microscope; and (5) a laser scanning confocal microscope. When correlated with respect to Profile 1, the CCF_max_ values were (2) 99.6%, (3) 92.1%, (4) 99.0%, and (5) 95.3%.

The comparisons were performed with a stylus instrument and three optical instruments, a coherence scanning interference microscope, a disk scanning confocal microscope, and a laser scanning confocal microscope. The profiles measured by the four instruments on the same area of a SRM bullet were compared with the profile of the virtual bullet signature standard traced by a stylus instrument on a bullet fired at ATF, which had served as the master for production of the SRM bullet replicas using a numerically controlled (NC) diamond turning machine. The comparison results, figure 44, show high agreement among the four techniques for 2D bullet profile signature comparisons. The CCF_max_ values were all higher than 90%. However, small differences in the fine details of the profile can be found in some measurements. The highest correlation value, CCF_max_ = 99.6%, came from the contact stylus instrument (Profile 2). This is understandable because the reference profile of the virtual standard (Profile 1) was established by the same stylus instrument. The comparison also shows high fidelity between the profile on the manufactured SRM bullets and the profile of the virtual standard used for the control of the NC diamond turning machine [56]. It is hard to find any differences in the fine details between these two profiles.

The measurement with the disk scanning confocal microscope (profile 4) also shows a high correlation value, CCF_max_ = 99.0%. There are small differences between the master profile 1 and both the profiles 5 and 3, which were measured by a laser scanning confocal microscope and an interferometric microscope, respectively. The CCF_max_ values are 95.3% and 92.1%. These differences might represent some instrument characteristics, for example, instrument noise, which could cause the difference in the fine profile details, and result in a slightly smaller CCF_max_ value. However, it should be emphasized that initial results for the confocal microscope were comparable to these values, and that the 99.0% CCF_max_ was obtained after optimization of the measurement conditions for the confocal microscope. Detailed measurement conditions can be found in Song *et al* [106].

### 6.2. Production QC

The SRM 2461 standard cartridge cases are replicated from an ATF master casing using the electro-forming technique [107]. All 127 replica cases have essentially the same topography. However, each replication can potentially degrade the master surface. In order to ensure that enough SRM casings were produced with virtually the same surface topography as the master, we tested the ‘decay factors’ for the replication process, that is, how fast the replication process itself would degrade the surface topography of the master. Test results showed that the decay factors were very small and that the topography of the SRM cases replicated from the same ATF master could be highly reproducible. During the testing process of the decay factors, we also observed that the parameter CCF_max_ is sensitive to surface defects, and therefore, could be used for production QC.

**Figure 45:**
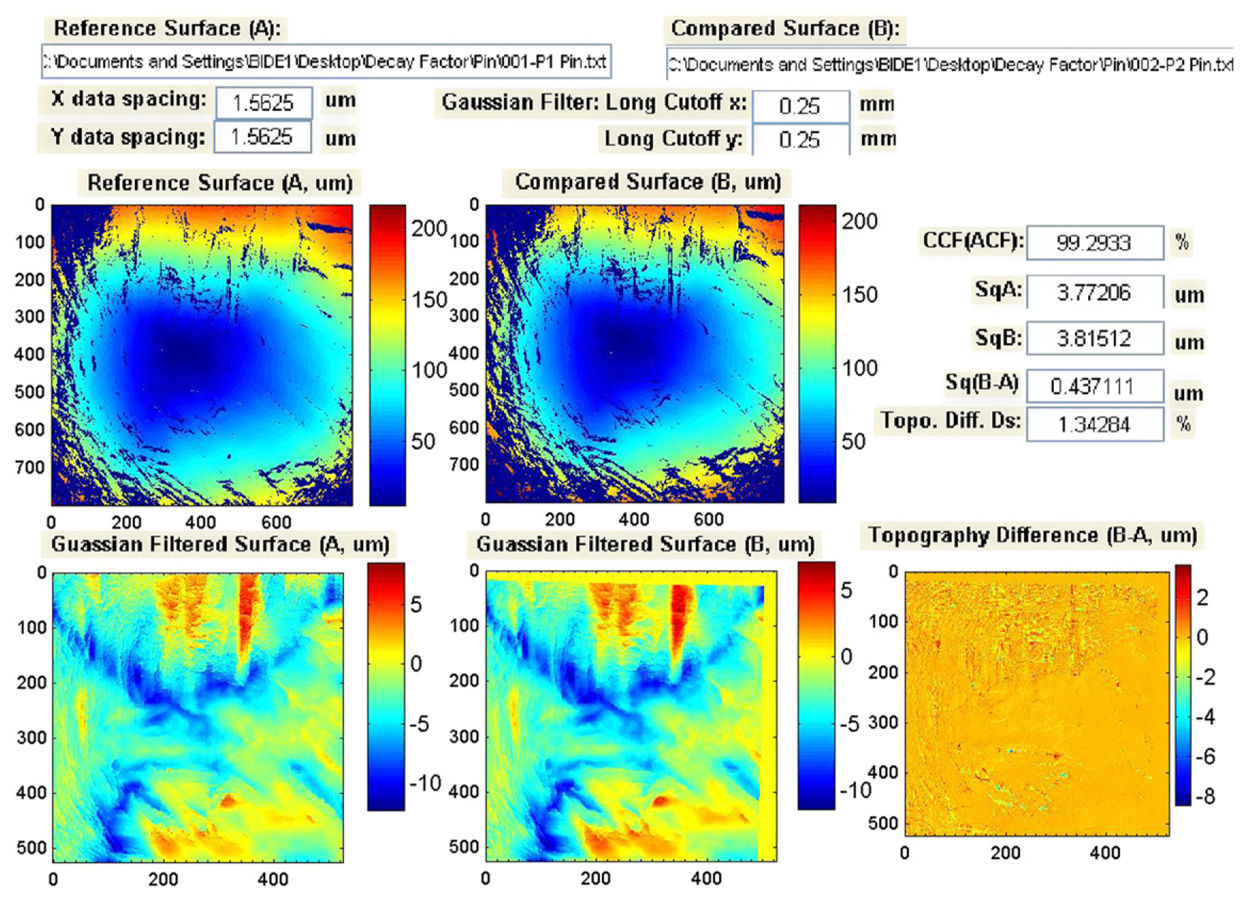
Correlation of topography images of firing pin impressions of prototype SRM casings 001 (top, left, used here as a reference) and 002 (top, right) [107]. The bottom row shows filtered images for 001 (left) and 002 (middle) casing and the topography difference (right). CCF_max_ = 99.29%, *D*_s_ = 1.34%.

**Figure 46:**
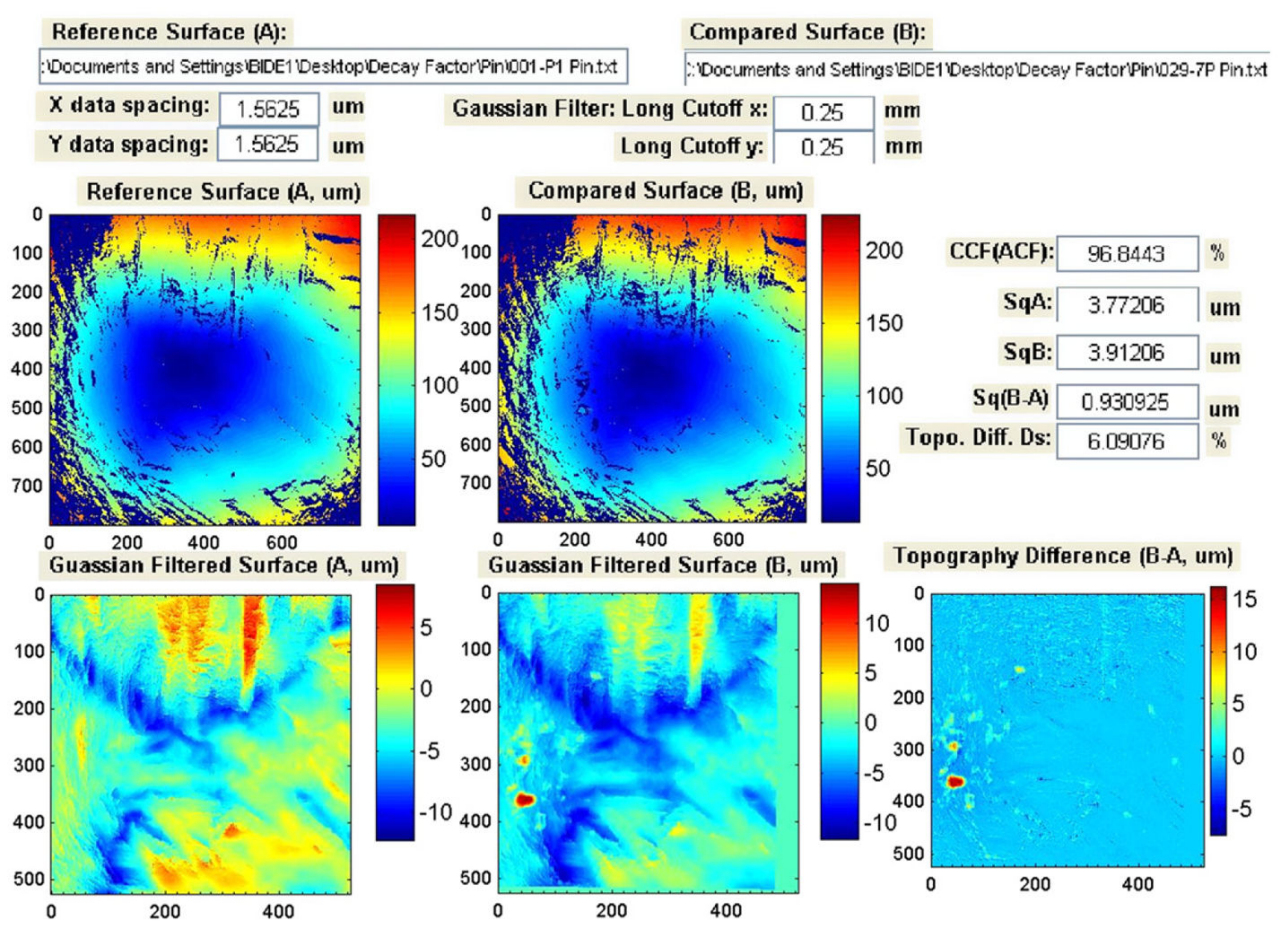
Correlation of topography images of firing pin impressions of prototype SRM casings 001 and 029. A surface defect on casing 029 can be seen in the bottom middle and right images. CCF_max_ = 96.60%, *D*_s_ = 5.79%.

Figures 45 and 46 show correlation results from the tests. In figure 45, the topography images of firing pin impressions for two prototype SRM cartridge cases, S/N 001 and S/N 002, are correlated. Neither image has significant surface defects, and CCF_max_ is equal to 99.29%. However, when the SRM S/N 029 cartridge case with a significant surface defect (figure 46) is correlated with the 001 cartridge case, the CCF_max_ value drops to 96.60%. From figure 46, it can be seen that there is a surface defect on the surface of the S/N 029 cartridge case. Comparison of figures 45 and 46 shows that the CCF_max_ value is sensitive to the surface defect, and therefore, could be useful for testing of surface defects in production QC.

## 7. Ongoing issues and opportunities

This review has been largely concentrated on the emerging field of surface topography measurements and analysis for ballistic surfaces and tool marks and aimed to provide useful information to surface metrologists and ballistics examiners in their common field of interest. Conventional optical microscopy is still more widely used in crime labs in the forms of comparison microscopes and automated imaging systems, such as IBIS [11]. In fact, a number of the statistical analysis methods discussed earlier can be applied to conventional optical images, not only to topography images and profiles. Topography measurement by optical methods is therefore a useful new tool for surface analysis in crime labs, one that is complementary to other methods. Whether, optical topography methods come to rival and outstrip the usefulness of conventional optical microscopy will likely depend on several factors:

### 7.1. Outliers and dropouts

Optical topography methods perform manipulations of the reflected optical signal from a surface, usually starting with measurement of a number of intensity images as the surface scans through its *z*-range in the field of the microscope. This signal manipulation leads to a reduced signal-to-noise ratio. Inevitably there are more unreliable data points in a topography image than a reflection microscopy image. Some points are dropouts or non-measured points that are spotted and identified as such by the measurement system software. Others are outliers, recorded data points that are clearly inaccurate and need to be corrected or ignored. A number of statistical methods have been used to discern and minimize the effect of these erroneous data points in the stored data. However, a standard approach for fired ballistics and tool marks may need to be defined in order to promote interoperability among topography images obtained with different optical methods.

### 7.2. Speed

All the optical topography methods discussed here must obtain lots of images, perhaps 1000, as the surface is scanned through different heights relative to the microscope housing. It is not surprising that topography imaging at the present level of computing technology is slower than conventional optical imaging. A NIBIN image of a breech face impression takes seconds to acquire after alignment and setup, whereas a topography image may take several minutes. Considering that databases like NIBIN contain on the order of a million images, converting current acquisition systems to topographical acquisition methods would be an expensive project. The daunting nature of this proposition is mitigated, however, by the outlook that high speed computing should continue to evolve at an impressive rate.

### 7.3. Expense

Currently topography measuring systems cost significantly more than conventional microscopes—roughly speaking, the one costs more than $100 000 and the other, less than $100 000. This differential is not likely to change. Both types of systems will likely rise or fall in price together. Because there are roughly 150 measurement systems in the NIBIN, the additional cost of new hardware to perform topography measurements is another daunting challenge to widespread use of topography methods by crime labs.

### 7.4. Uncertainty

Topography methods coupled with advanced statistical analyses have finally provided an opportunity to address the question of uncertainty in firearm and tool mark identifications. Several case studies we discussed here have resulted in the calculation of an error rate for identification and exclusion of matching surfaces fired by the same firearm. In some cases those error rates have been impressively small even for consecutively produced barrels or slides or tools. However, the most advanced work has so far been performed on small databases or on small collections of firearms or other materials. Scaling up the models to large databases like the NIBIN and adjusting the statistical model to produce believable error rates for real criminal cases is a major challenge and a major opportunity for researchers. Once accomplished, such a development will pave the way to calculating error rates for firearms identification for real court cases, first as an independent approach to support conclusions drawn by firearms experts using comparison microscopes, and possibly afterwards, to stand on its own as admissible evidence in court in a manner similar to DNA evidence.

The implication from figures 20, 26, 29, 30, 35, 37, 42, and 43 is that separation of populations is a key factor. How does one devise measurement and analysis methods that provide a clear separation between populations of matching and non-matching pairs of images that will be appropriate for large populations with many sources of variability among images? Along this line, parameters to quantify the fractional overlap between population distributions and between their fitted models, perhaps even a single accepted one, say, similar to the *empirical error rate* parameter described by Bachrach *et al* [31] would be most useful.

We have been discussing the merits of surface topography measurement and analysis for use in crime labs, and in some places we have highlighted the relative merits of topography measurement and automated optical microscopy. Until now, we have assumed that the main application is criminal investigations involving firearms and toolmark identification. Lurking behind these issues is the possibility that either of these methods might be used as part of expert evidence submitted by firearms examiners in court cases. Traditionally, the only type of admissible court evidence or testimony has been derived from the experts use of comparison microscopy as discussed in ‘ section 1.1. That type of evidence does not lend itself to quantitative statements of error rates. Error rates, however, can be calculated for DNA analysis, and it is the fond hope of judges that quantitative error rates can accompany evidence from other forensic science fields including firearms analysis [88]. The quantitative results for error rate that are possible with topography measurement and analysis make this a promising method that can eventually be used to support or modify the conclusions of the firearms and toolmark examiners, otherwise limited to the traditional comparison techniques.

## List of acronyms

ACCF: Areal cross correlation function
AFTE: Association of Firearm and Toolmark Examiners
ASCLD/LAB: American Society of Crime Laboratory Directors/Laboratory Accreditation Board
ATF: Bureau of Alcohol, Tobacco, Firearms, and Explosives
CCF: Cross-correlation function
CMC: Congruent matching cells
CMS: Consecutive matching striae
CSI: Coherence scanning interferometry
CV: Cross validation
DNA: Deoxyribonucleic acid
DSCM: Disk scanning confocal microscopy
DWT: Discrete wavelet transform
GPU: Graphical processing unit
HOO-CV: Hold-one-out cross-validation
IBIS: Integrated ballistics identification system
ISO: International Organization for Standardization
KDE: Kernel density estimate
KM: Known matching
KNM: Known non-matching
LEA: Land engraved area
LED: Light emitting diode
LSCM: Laser scanning confocal microscopy
NA: Numerical aperture
NC: Numerically controlled
NIBIN: National Integrated Ballistic Information Network
NIST: National Institute of Standards and Technology
PAM: Programmable array microscope
PCA: Principal component analysis
QC: Quality control
SI: The international system of units
SRM: Standard Reference Material
SVM: Support vector machine
VIM: International Vocabulary of Metrology
